# Impact of platinum-based chemotherapy and CTLA-4 inhibition on acquired resistance to first-line anti-PD-1/PD-L1 agents in non-small cell lung cancer: a systematic review and reconstructed individual patient data analysis

**DOI:** 10.1016/j.eclinm.2025.103482

**Published:** 2025-09-02

**Authors:** Sara Oresti, Fabio Salomone, Antonio Nuccio, Francesca Rita Ogliari, Silvia Teresa Riva, Ludovica Mollica, Alessandra Bulotta, Maria Grazia Viganò, Francesco Maria Venanzi, Francesco Passaretti, Ludovica Papotto, Anna Di Lello, Michele Ferrara, Giorgia Foggetti, Giuseppe Damiano, Alberto Servetto, Raffaele Califano, Massimo Di Maio, Biagio Ricciuti, Alessio Cortellini, Martin Reck, Michela Cinquini, Valter Torri, Giuseppe Viscardi, Roberto Ferrara

**Affiliations:** a Department of Medical Oncology, IRCCS Ospedale San Raffaele, Milan, Italy; b Department of Clinical Medicine and Surgery, University of Naples Federico II, Naples, Italy; c Università Vita-Salute San Raffaele, Milan, Italy; d Division of Experimental Oncology, IRCCS San Raffaele Scientific Institute, Milan, Italy; e Department of Medical Oncology, The Christie NHS Foundation Trust and Division of Cancer Sciences, The University of Manchester, United Kingdom; f Department of Oncology, University of Turin, AOU Città della Salute e della Scienza di Torino, Turin, Italy; g Dana-Farber Cancer Institute, Boston, United States; h Department of Medicine and Surgery, Università Campus Bio-Medico di Roma, Roma, Italy; i LungenClinic, Airway Research Center North, German Center for Lung Research, Grosshansdorf, Germany; j Clinical Oncology Department, Istituto di Ricerche Farmacologiche Mario Negri IRCCS, Milan, Italy; k Department of Pneumology and Oncology, PO Monaldi-AORN Ospedali dei Colli, Naples, Italy; l Medical Oncology, Fondazione Policlinico Universitario Campus Bio-Medico, Rome, Italy; m Department of Surgery and Cancer, Hammersmith Hospital Campus, Imperial College London, London, UK

**Keywords:** Acquired resistance, Platinum-based chemotherapy, CTLA-4 inhibitors, Immunotherapy, Non-small cell lung cancer

## Abstract

**Background:**

Acquired resistance is defined as disease progression following an initial response to immune checkpoint inhibitors (ICI). The impact of adding platinum-based chemotherapy (PCT) or anti-CTLA-4 agents to PD-(L)-1 inhibitors on acquired resistance is currently unknown.

**Methods:**

Systematic research by January 31, 2025 identified randomized clinical trials (RCTs) evaluating first-line ICI as monotherapy (mono-ICI) or in combination with PCT (mono-ICI + PCT), CTLA-4 inhibitors (combo-ICI), or both (combo-ICI + PCT) in metastatic non-small-cell lung cancer (NSCLC). RCTs reporting duration of response (DoR) data were eligible. Acquired resistance rates at 6 and 12 months were estimated from DoR curves. Aggregate data based on type of regimens were reported with risk ratio (RR) and pooled by random effect model. Primary and secondary endpoints were respectively the indirect comparison of acquired resistance risk between PCT-containing versus PCT-free regimens and between anti-CTLA-4 containing versus anti-CTLA-4 free regimens, respectively. Time-to-event outcomes were retrieved from Kaplan–Meier (KM) curves using individual patient data (IPD) and compared by log rank tests. This study was registered to the PROSPERO online platform (CRD42025639320).

**Findings:**

Nineteen RCTs were included. 6- and 12-months acquired resistance rates were 16.5%–34.1% (mono-ICI), 26.4%–47.8% (mono-ICI + PCT), 19.0%–33.0% (combo-ICI), and 28.4%–47.1% (combo-ICI + PCT). A higher risk of acquired resistance was suggested from indirect comparisons of mono-ICI + PCT versus mono-ICI (12-months RR: 1.46, 95% CI 1.23–1.75) and of combo-ICI + PCT versus combo-ICI (12 months RR: 1.36, 95% CI 1.06–1.73). Using the reconstructed patient-level data, median DoR was 5–7 months significantly shorter with PCT-containing compared to PCT-free regimens. The addition of anti-CTLA-4 agents did not impact on acquired resistance.

**Interpretation:**

Although insufficient RCTs reported acquired resistance rates stratified by specific factors influencing DoR (i.e., PD-L1, TMB) for such analyses to be included in this report, the increased acquired resistance risk observed with PCT-containing regimens highlights the potential value of tailoring first-line treatment strategies in NSCLC on the basis of individual risk of resistance to ICI.

**Funding:**

This research did not receive any specific grant from funding agencies.

**Research in context**

**Evidence before this study**

To explore the available evidence, we searched PubMed and EMBASE up to January 31, 2025, using terms related to “lung cancer” and “immune checkpoint inhibitors.” We identified trials evaluating ICIs in non-small-cell lung cancer (NSCLC) patients treated in the first-line setting. Up to 64% of patients who initially respond to immune checkpoint inhibitors (ICIs) develop acquired resistance within 5 years, yet the biological and clinical determinants of acquired resistance remain poorly defined. Platinum-based chemotherapy (PCT) is known to reduce early mortality when combined with anti–PD-1/PD-L1 agents, but its impact on acquired resistance is uncertain. Similarly, the role of CTLA-4 inhibition in modulating acquired resistance risk has not been systematically investigated.

**Added value of this study**

In the present reconstructed individual patient’s data analysis, we found that the addition of PCT increases by ≃40% and ≃50% the relative risk of developing acquired resistance within 6- or 12-months, respectively, resulting in a 5–7 months lower median DoR with PCT-containing compared to no PCT-regimens. No effect of CTLA-4 inhibition was found on acquired resistance risk. This work fills a gap in the literature by providing quantitative estimates of acquired resistance risk associated with current first-line regimens and highlighting the trade-offs of PCT-containing strategies.

**Implications of all the available evidence**

These data suggest a more thoughtful patient selection before first-line treatment initiation, offering a PCT-containing regimen mainly to patients at high risk of primary resistance and sparing PCT in other cases in order to potentially delay the emergence of acquired resistance. Supporting the hypothesis of a negative impact of PCT on acquired resistance, the present analysis paves the way for future translational studies, exploring the genomic and immunological landscapes associated with acquired resistance to combination treatment strategies.

## Introduction

Immune checkpoint inhibitors (ICIs) revolutionised the treatment of non-small-cell lung cancer (NSCLC),^1^ however, aside from PD-L1 expression level, reliable biomarkers to choose between first-line single-agent PD-1/PD-L1 inhibitors (mono-ICI), anti-PD-(L)-1 agents in combination with CTLA-4 inhibitors (combo-ICI) and ICI-platinum-based chemotherapy (PCT) combinations are currently lacking. Indeed, PD-L1 expression and tumour mutational burden (TMB) have several limitations due to spatial and temporal heterogeneity,^2^ threshold levels, and technical and biological issues.^3^ Therefore, it is currently challenging to predict who are the patients with long-term benefit with mono-ICI and who will need a treatment escalation with the addition of PCT or anti-CTLA-4 agents. We previously reported that the addition of PCT to first-line ICI may prevent primary resistance in NSCLC patients enrolled in clinical trials,^4^ by halving the early mortality rate within the first 3 months of treatment (8% with mono-ICI–PCT versus 16% with mono-ICI). On the other hand, no effect on the early death risk was observed with the addition of CTLA-4 inhibitors to anti-PD-1/PD-L1 agents.^4^

So far, the impact of combination strategies on acquired resistance to anti PD-1/PD-L1 agents in NSCLC patients is unknown. In recent years, acquired resistance has gained increasing attention by the scientific community, due to the need of identifying novel treatment strategies able to delay progression to first line regimens or improve outcomes in second line setting.

The Society for Immunotherapy of Cancer has developed criteria to define primary and acquired resistance to ICIs across cancer types with the latter described as radiological progression (progressive disease) after an initial partial response or stable disease lasting for at least 6 months, with a confirmatory scan required for progressive disease assessment.^5^

Schoenfeld et al. have proposed different criteria for acquired resistance which was defined as progressive disease after an initial partial response (stable disease was not allowed in the criteria), with no temporal threshold from the last ICI dose.^6^ According to Schoenfeld’s criteria, by using Kaplan–Meier curves of duration of response (DoR) from clinical trials with mono-ICI in second line setting, acquired resistance at 5 years occurred in 64% of initially responding NSCLC patients.^7^ Similarly, in a retrospective study, acquired resistance was reported in 46% of NSCLC patients responding to first-line ICI. Of note, in 42% of patients, acquired resistance occurred in the first 12 months after ICI initiation and it was associated with PD-L1 expression ≥ 50% and lower rate of liver, bone, and lung metastases compared to primary ICI resistance.^8^^,^^9^ So far, acquired resistance has not been systematically explored in first line setting in NSCLC patients enrolled in clinical trials, and the role of PCT or CTLA-4 inhibitors in influencing acquired resistance upon PD-1/PD-L1 is currently unknown. In the present manuscript, we conducted a systematic review and a meta-analysis using both aggregate data and individual patient data obtained by graphical reconstruction of Kaplan–Meier curves, in order to assess acquired resistance rate with first line mono-ICI, combo-ICI and ICI–PCT combinations across randomized clinical trials in untreated NSCLC patients. To define the specific role of the addition of PCT and/or CTLA-4 inhibitors to anti-PD-1/PD-L1 agents in modulating acquired resistance rate, we performed indirect comparisons of risk ratios and survival outcomes between mono-ICI, mono-ICI–PCT combo-ICI and combo-ICI–PCT combinations.

## Methods

Preferred Reporting Items for Systematic Reviews and Meta-Analysis (PRISMA) guidelines were utilized to report this systematic review and meta-analysis.^10^ The full protocol was registered to the PROSPERO online platform (CRD42025639320).

### Search strategy and selection criteria

Systematic literature search in PubMed-indexed journals and EMBASE database was conducted for all articles published up to January 31, 2025. The research string is reported in Supplementary Table S1. Studies were considered eligible for further analysis if all these conditions were satisfied: 1) phase II or phase III randomized clinical trials (RCTs) testing PD-1/PD-L1 and/or CTLA-4 inhibitors in advanced or metastatic NSCLC; 2) ICI administered in first line setting; 3) available data on DoR; 4) studies written in English. Trials investigating concomitant local treatments were excluded from our analysis, as well as brief communications, study protocols or designs and subgroup or post-hoc analysis.

The primary endpoint of this meta-analysis was to compare the risk of acquired resistance between PCT-containing (mono-ICI–PCT, combo-ICI–PCT) versus PCT-free (mono-ICI or combo-ICI) ICI regimens.

The secondary endpoint was to compare the risk of acquired resistance between CTLA-4 inhibitors containing (combo-ICI and combo-ICI–PCT) versus CTLA-4 inhibitors free (mono-ICI and mono-ICI–PCT) regimens.

Two authors (S.O. and F.S.) independently evaluated eligibility criteria of all screened articles using titles, abstracts and full texts. Conflicts were resolved by consensus with another author (A.N.). For each trial, acronyms, outcomes of interest such as median DoR, overall response rate (ORR), use of blinded independent central review (BICR) for response assessment, PD-L1 expression or blood tumour mutational burden (bTMB) of the population with available DoR, type of regimens and median follow-up time were collected. Risks of bias were assessed by two authors (S.O. and G.V.) using the Cochrane risk of Bias tool (RoB)^11^ and discrepancies were resolved by a third author (R.F.). Funnel plots to assess publication bias were reported if there were at least 10 included studies.

### Data analysis

Based on Schoenfeld’s criteria, patients, achieving a partial response were retrieved from the DoR Kaplan–Meier (KM) curves and the risk of acquired resistance was analysed accordingly. Graphical reconstructive algorithm was used to estimate time-to-event outcome of interest from reported KM curves, according to Guyot^12^ and Liu.^13^ 6-months and 12-months acquired resistance, as well as 6-months and 12-months censored patients were retrieved from reconstruction of both experimental and control arms, by summing the total number of events at each cut-off. Cumulative KM curves were built to compare DoR between mono-ICI–PCT versus mono-ICI and combo-ICI–PCT versus combo-ICI (primary endpoint) and between mono-ICI–PCT versus combo-ICI–PCT and mono-ICI versus combo-ICI (secondary endpoint). Survival analyses were performed by Cox-proportional hazard model and log-rank test. Risk ratios (RR) of acquired resistance at 6-months and 12-months in each group (mono-ICI, mono-ICI–PCT, combo-ICI, combo-ICI–PCT) were compared by a random effect model due to potential heterogeneity. Cochrane Q-test was used to assess statistical heterogeneity among subgroups with a significant threshold set at alpha = 0.1. Inconsistency among subgroups was quantified by the I-squared statistic with I-square > 50% judged as significant heterogeneity. Indirect comparisons between mono-ICI–PCT versus mono-ICI, combo-ICI–PCT versus combo-ICI, combo-ICI–PCT versus mono-ICI–PCT and combo-ICI versus mono-ICI were performed as previously described by Bucher and Glenny.^14^^,^^15^ In detail, using the chemotherapy-based control arm as the common comparator, cumulative RR estimation was obtained by logarithmically subtracting the independent RR estimates from one set of studies from those of another set of studies. The cumulative variance was obtained by summing the two variances. Censoring imbalance between groups was assessed by the reverse Kaplan–Meier method.^16^ Statistical significance was set at p-value ≤ 0.05. All the statistical analyses were conducted using IBM SPSS statistics (version 29.1.0) and the RevMan software (version 5.4).

Prior to conducting the pooled analysis, a quality check of the KM reconstruction was assessed. The reconstructed KM curves for DoR were compared to the original published KM curves. The evaluation was based on a graphical comparison of curve shape, as well as marginal median DoR and number of patients at risk.

### Ethics

Ethics approval is not required for this systematic review of published literature.

### Role of the funding source

There was no funding source for this study.

## Results

**Fig. 1:**
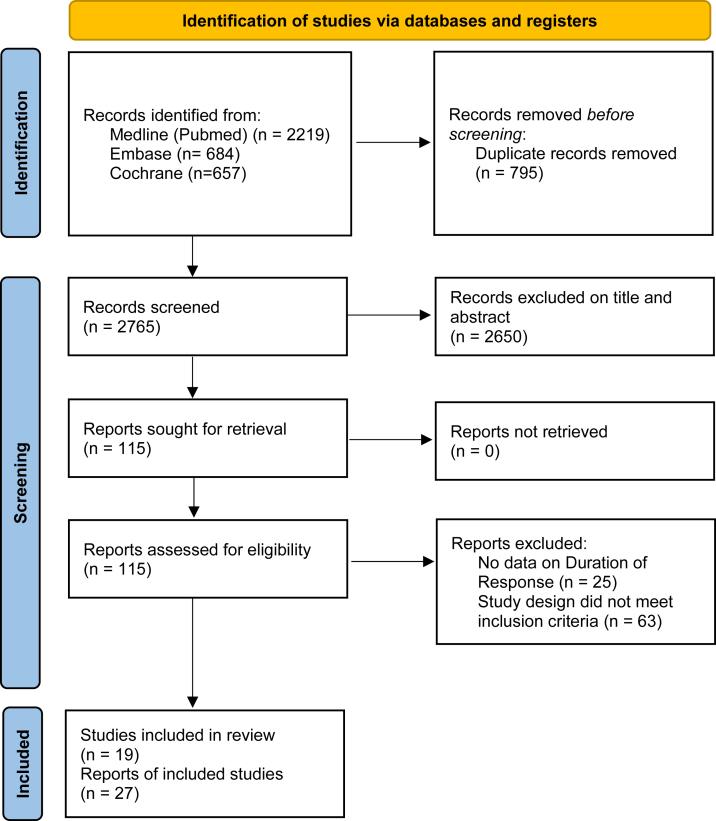
PRISMA diagram.

Literature research identified 3560 records up to January 31, 2025 (Fig. 1). The research string is reported in Supplementary Table S1. 19 RCTs were included. Main characteristics of included RCTs are summarized in Table 1. Clinical characteristics of patients, screening failure rate and stratification factors of the studies were reported in Supplementary Table S2.

**Table 1:** Characteristics of included studies.

Trial name	Patient	EXP arm	Type of ICI	CTRL arm	ORR endpoint	ORR EXP n	ORR CTRL n	ORR EXP %	ORR CTRL %	BICR	DoR endpoint	mDoR EXP	mDoR CTRL	mFU DoR
KEYNOTE042^c^	≥1% PD-L1	Anti-PD-1	Pembrolizumab	PCT	Exploratory	174/637	169/637	27%	27%	Yes	Exploratory	20.2 (16.6–NR)	8.3 (6.5–11.1)	12.8
KEYNOTE042^c^	≥20% PD-L1	Anti-PD-1	Pembrolizumab	PCT	Exploratory	138/413	117/405	33%	29%	Yes	Exploratory	20.2 (16.3–NR)	8.3 (6.4–13.4)	12.8
KEYNOTE042^c^	≥50% PD-L1	Anti-PD-1	Pembrolizumab	PCT	Exploratory	118/299	96/300	39%	32%	Yes	Exploratory	20.2 (16.6–NR)	10.8 (6.1–13.4)	12.8
MYSTIC^c^	Any PD-L1 (bTMB ≥ 20 mut/Mb)	Anti-PD-L1	Durvalumab	PCT	Exploratory	23/77	15/70	30%	21%	Yes	Exploratory	NR (NR–NR)	4.1 (3.0–4.3)	12.0^b^
MYSTIC^c^	Any PD-L1 (bTMB < 20 mut/Mb)	Anti-PD-L1	Durvalumab	PCT	Exploratory	43/209	58/185	20%	31%	Yes	Exploratory	NR (5.9–NR)	4.1 (2.8–5.6)	12.0^b^
MYSTIC^c^	Any PD-L1 (bTMB ≥ 20 mut/Mb)	Anti-PD-L1 + anti-CTLA4	Durvalumab + tremelimumab	PCT	Exploratory	31/64	15/70	48%	21%	Yes	Exploratory	NR (NR–NR)	4.1 (3.0–4.3)	12.0^b^
MYSTIC^c^	Any PD-L1 (bTMB < 20 mut/Mb)	Anti-PD-L1 + anti-CTLA4	Durvalumab + tremelimumab	PCT	Exploratory	34/204	58/185	17%	31%	Yes	Exploratory	11.1 (5.6–NR)	4.1 (2.8–5.6)	12.0^b^
PEARL	≥25% PD-L1	Anti-PD-L1	Durvalumab	PCT	Secondary	116/335	92/334	34%	27%	No	Secondary	14.0 (10.2–22.6)	4.8 (4.2–6.9)	53.5
PEARL	≥50% PD-L1	Anti-PD-L1	Durvalumab	PCT	Secondary	95/247	75/246	38%	30%	No	Secondary	14.3 (9.7–24.4)	4.5 (4.2–5.7)	53.5
Checkmate227	Any PD-L1	Anti-PD-1 + anti-CTLA4	Nivolumab + ipilimumab	PCT	Secondary	195/583	161/583	33%	28%	Yes	Secondary	19.6 (16.1–28.6)	5.8 (5.4–6.9)	29.3
CheckMate227 part 1	≥1% PD-L1	Anti-PD-1 + anti-CTLA4	Nivolumab + ipilimumab	PCT	Secondary	144/396	118/397	37%	30%	Yes	Secondary	24.5 (15.5–33.9)	6.7 (5.6–7.6)	29.3
CheckMate227 part 1	≥1% PD-L1	Anti-PD-1	Nivolumab	PCT	Secondary	109/396	118/397	28%	30%	Yes	Secondary	15.5 (12.5–20.8)	6.7 (5.6–7.6)	29.3
Checkmate227 part 1	≥50% PD-L1	Anti-PD-1 + anti-CTLA4	Nivolumab + ipilimumab	PCT	Secondary	93/205	68/192	44%	35%	Yes	Secondary	31.8 (18.7–NR)	5.8 (4.5–6.9)	29.3
Checkmate227 part 1	≥50% PD-L1	Anti-PD-1	Nivolumab	PCT	Secondary	79/214	68/192	37%	35%	Yes	Secondary	17.5 (13.5–31.0)	5.8 (4.5–6.9)	29.3
CheckMate227 part 1	<1% PD-L1	Anti-PD-1 + PCT	Nivolumab	PCT	Secondary	67/177	43/186	38%	23%	Yes	Secondary	8.3 (5.9–9.4)	4.8 (3.7–5.8)	29.3
CheckMate227 part 1	<1% PD-L1	Anti-PD-1 + anti-CTLA4	Nivolumab + ipilimumab	PCT	Secondary	51/187	43/186	27%	23%	Yes	Secondary	19.4 (12.4–33.2)	4.8 (3.7–5.8)	29.3
CheckMate227 part 2	Any PD-L1	Anti-PD-1 + PCT	Nivolumab	PCT	Secondary	194/377	114/378	51%	30%	Yes	Exploratory	10.6 (8.3–13.1)	6.9 (5.1–9.3)	19.5
CameL	Any PD-L1	Anti-PD-1 + PCT	Camrelizumab	PCT	Secondary	113/205	68/207	55%	33%	Yes	Secondary	15.5 (10.6–25.4)	10.3 (6.8–14.0)	11.9
CameLSQ	Any PD-L1	Anti-PD-1 + PCT	Camrelizumab	PCT	Secondary	125/193	72/196	65%	36%	Yes	Secondary	13.1 (9.3–15.7)	4.4 (4.2–4.9)	13.5^a^
NEPTUNE	bTMB ≥ 20 mut/Mb	Anti-PD-L1 + anti-CTLA4	Durvalumab + tremelimumab	PCT	Secondary	19/69	26/60	27%	43%	No	Secondary	11.6 (5.6–21.5)	4.2 (3–6.9)	32.9
KEYNOTE598	≥50% PD-L1	Anti-PD-1 + anti-CTLA4	Pembrolizumab + ipilimumab	Anti-PD-1	Secondary	129/282	129/281	45%	45%	Yes	Secondary	16.1 (1.1–26.0)	17.3 (2.0–29.4)	20.6
POSEIDON	Any PD-L1	Anti-PD-L1 + anti-CTLA4 + PCT	Durvalumab + tremelimumab	PCT	Secondary	130/335	81/332	39%	24%	Yes	Secondary	9.5 (7.2–NE)	5.1 (4.4–6.0)	10.5^b^
POSEIDON	Any PD-L1	Anti-PD-L1 + PCT	Durvalumab	PCT	Secondary	137/330	81/332	41%	24%	Yes	Secondary	7 (5.7–9.9)	5.1 (4.4–6.0)	10.5^b^
CheckMate9LA	Any PD-L1	Anti-PD-1 + anti-CTLA4 + PCT	Nivolumab + ipilimumab	PCT	Hierarchical secondary	137/361	91/358	38%	25%	Yes	Hierarchical secondary	12.4 (8.7–20.2)	5.6 (4.4–7.1)	64.9
Checkmate9LA	<1% PD-L1	Anti-PD-1 + anti-CTLA4 + PCT	Nivolumab + ipilimumab	PCT	Hierarchical secondary	42/135	26/129	31%	20%	Yes	Hierarchical secondary	17.5 (6.9–37.8)	4.3 (2.8–7.1)	64.9
Checkmate9LA	≥1% PD-L1	Anti-PD-1 + anti-CTLA4 + PCT	Nivolumab + ipilimumab	PCT	Hierarchical secondary	87/204	57/204	43%	28%	Yes	Hierarchical secondary	11.8 (8.6–20.3)	5.6 (4.3–8.0)	64.9
Checkmate9LA^c^	1–49% PD-L1	Anti-PD-1 + anti-CTLA4 + PCT	Nivolumab + ipilimumab	PCT	Hierarchical secondary	49/128	26/106	39%	24%	Yes	Hierarchical secondary	10.8 (6.9–19.4)	7.0 (3.9–14.5)	64.9
Checkmate9LA^c^	≥50% PD-L1	Anti-PD-1 + anti-CTLA4 + PCT	Nivolumab + ipilimumab	PCT	Hierarchical secondary	38/76	31/98	50%	31%	Yes	Hierarchical secondary	26.0 (8.6–33.3)	5.4 (3.9–10.9)	64.9
NIPPON	Any PD-L1	Anti-PD-1 + anti-CTLA4 + PCT	Nivolumab + ipilimumab	Anti-PD-1 + PCT	Secondary	76/139	89/136	55%	65%	No	Secondary	11.3 (6.2–15.2)	8.0 (5.6–11.4)	15.3
CHOICE-01	Any PD-L1	Anti-PD-1 + PCT	Toripalimab	PCT	Secondary	203/309	72/156	66%	46%	Yes	Secondary	8.4 (6.8–12.9)	4.2 (4.0–5.6)	16.2
EmpowerLUNG3	Any PD-L1	Anti-PD-1 + PCT	Cemiplimab	PCT	Secondary	136/312	34/154	44%	22%	Yes	Secondary	16.4 (13.1–18.9)	7.3 (4.2–11.3)	16.3^a^
GEMSTONE302	Any PD-L1	Anti-PD-1 + PCT	Sugemalimab	PCT	Secondary	203/320	64/159	63%	40%	Yes	Secondary	9.9 (8.6–13.2)	4.4 (3.5–6.1)	17.8
GEMSTONE302^c^	<1% PD-L1	Anti-PD-1 + PCT	Sugemalimab	PCT	Secondary	64/124	25/64	52%	39%	Yes	Secondary	7.5 (5.5–11.7)	3.7 (2.9–6.3)	17.8
GEMSTONE302^c^	≥1% PD-L1	Anti-PD-1 + PCT	Sugemalimab	PCT	Secondary	139/196	39/95	71%	45%	Yes	Secondary	11.5 (9.4–16.6)	5.1 (3.5–6.7)	17.8
IMpower130	Any PD-L1	Anti-PD-L1 + PCT	Atezolizumab	PCT	Secondary	220/447	72/226	49%	32%	Yes	Secondary	8.4 (6.9–11.8)	6.1 (5.5–7.9)	18.5^a^
KEYNOTE021	Any PD-L1	Anti-PD-1 + PCT	Pembrolizumab	PCT	Primary	35/60	21/63	58%	33%	Yes	Secondary	36.3 (1.4–49.3)	22.8 (2.8–47.2)	10.6
KEYNOTE189	Any PD-L1	Anti-PD-1 + PCT	Pembrolizumab	PCT	Secondary	198/410	41/206	48%	20%	Yes	Secondary	12.7 (1.1–68.3)	7.1 (2.4–31.5)	10.5
KEYNOTE407	Any PD-L1	Anti-PD-1 + PCT	Pembrolizumab	PCT	Secondary	173/278	109/281	62%	39%	Yes	Secondary	9 (1.3–61.5)	4.9 (1.3–58.6)	7.8

PD-L1: programmed death-ligand 1, PD-1: programmed cell death protein 1, CTLA4: Cytotoxic T-lymphocyte-associated protein 4, PCT: platinum based chemotherapy, bTMB: blood tumour mutational burden, ICI: immunecheckpoint inhibitors, CTRL: control, EXP: experimental, ORR: overall response rate, mDoR: median duration of response, BICR: blinded independent central review, mFU: median follow up.

a mFU for the experimental arm.

b Calculated using the cutoff date.

c Kaplan–Meier plots for the PD-L1/TMB subgroups were not available from the original publication.

Eligible RCTs were divided into four types according to experimental treatment regimens: i) first line anti PD-1/PD-L1 (mono-ICI) in combination with PCT (12 RCTs); ii) first line anti PD-1/PD-L1 + anti CTLA-4 (combo-ICI) in combination with PCT (3 RCTs); iii) first line anti PD-1/PD-L1 (mono-ICI) (4 RCTs); and iv) first line anti PD-1/PD-L1 + anti CTLA-4 (combo-ICI) (3 RCTs).

No study employing a CTLA-4 inhibitor as monotherapy or in combination with PCT (without anti-PD-1/PD-L1 agents) was eligible in the mono-ICI or mono-ICI + PCT categories, respectively. DoR was a secondary or exploratory endpoint in 16 and 3 RCTs, respectively. BICR for response assessment was applied in 16 RCTs. Six RCTs (CheckMate 227, KEYNOTE 042, KEYNOTE 598, CheckMate 9LA, PEARL and GEMSTONE 302) reported DoR according to different PD-L1 expression categories. However, only 4 studies (CheckMate 227. KEYNOTE 598, Checkmate 9LA, PEARL) had KM of DoR stratified according to PD-L1 expression levels. One RCT (NEPTUNE) reported KM of DoR according to bTMB.

Risk of bias was assessed using available information in full articles or in protocol. All RCTs were at low risk of selection bias as the random sequence generation method was adequately reported. High risk of performance, detection or attrition bias was identified in ten, four and one RCT respectively (Supplementary Table S3). Regarding reporting bias, one trial was judged at high risk, and another at unclear risk. Supplementary Figs. S1 and S2 showed risk of bias summary overall and for each included RCT, respectively. Visual inspection of funnel plot showed no risk of publication bias for the comparison mono-ICI + PCT versus PCT, while it was not possible to evaluate publication bias for other comparisons due to the low number of included studies (Supplementary Fig. S3).

The addition of ICI reduced acquired resistance at 6 and 12 months compared to PCT alone. At 6 months, acquired resistance rates upon mono-ICI + PCT or combo-ICI + PCT compared to PCT were 26.4% (478/1804) versus 48.5% (384/791) and 28.4% (76/267) versus 52.9% (91/172), respectively (Supplementary Fig. S4A). A statistically significant reduction in the risk of acquired resistance was observed for both mono-ICI + PCT (RR: 0.56, 95% CI 0.50–0.64, I2: 24%, 12 RCTs, 2595 participants) and combo-ICI + PCT (RR: 0.54, 95% CI 0.43–0.68, I2: 0%, 2 RCTs, 439 participants) compared to PCT (Supplementary Fig. S5A and B). Similarly, acquired resistance rates at 6 months were lower with mono-ICI (16.5% versus 44.0%; 77/465 versus 199/452) and combo-ICI (19.0% versus 50.7%; 53/279 versus 132/260) compared to PCT (Supplementary Fig. S4A) and a statistically significant reduction in the risk of acquired resistance was reported for both mono-ICI (RR: 0.38, 95% CI 0.30–0.47, I2: 0%, 4 RCTs, 917 participants) and combo-ICI compared to PCT (RR: 0.39, 95% CI 0.30–0.51; I2: 0%, 3 RCTs, 539 participants) (Supplementary Fig. S5C and D).

**Fig. 2:**
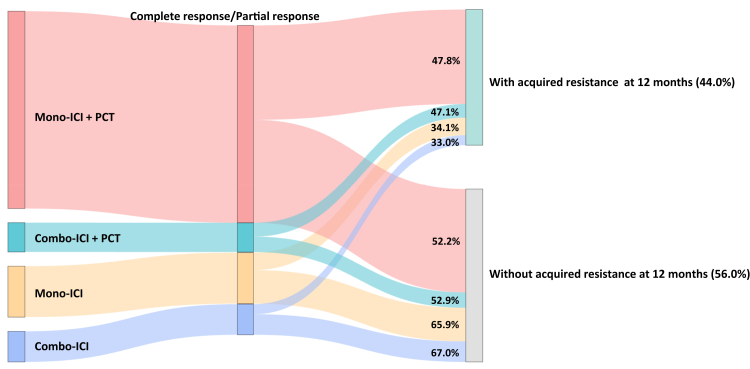
Alluvial plot of acquired resistance rates at 12 months according to treatment regimens (17 RCTs). ICI, immune checkpoint inhibitors; PCT, platinum-based chemotherapy.

At 12 months, the difference in acquired resistance rates remained similar to what reported at 6 months for both mono-ICI–PCT (47.8%; 862/1804) and combo-ICI–PCT (47.1%; 126/267) (Fig. 2) compared to PCT (66.2%–69.7%; 524/791-120/172) (Fig. 2, Supplementary Fig. S4B), with a statistically significant reduction in the risk of acquired resistance (mono-ICI versus PCT: RR: 0.72, 95% CI 0.65–0.80, I2: 53%, 12 RCTs, 2595 participants; combo-ICI versus PCT: RR: 0.68, 95% CI 0.58–0.79, I2: 0%, 2 RCTs, 439 participants) (Supplementary Fig. S6A and B). Similarly, acquired resistance rate at 12 months was twice lower for mono-ICI (34.1%; 159/465) and combo-ICI (33.0%; 92/279) (Fig. 2) compared to PCT (68.3%–67.7%; 309/452-176/260) (Fig. 2, Supplementary Fig. S4B), with a statistically significant reduction in the risk of acquired resistance for ICI including regimens (mono-ICI versus PCT: RR: 0.49, 95% CI 0.43–0.57, I2: 0%, 4 RCTs, 917 participants; combo-ICI versus PCT: RR 0.50, 95% CI 0.41–0.60, 3 RCTs, I2: 0%, 539 participants) (Supplementary Fig. S6C and D). Alluvial plot in Fig. 2 provides graphical comparison of acquired resistance rates according to ICI regimen (mono-ICI + PCT, combo-ICI + PCT, mono-ICI, combo-ICI). 6- and 12-months acquired resistance rates for each trial are reported in Supplementary Table S4.

**Fig. 3:**
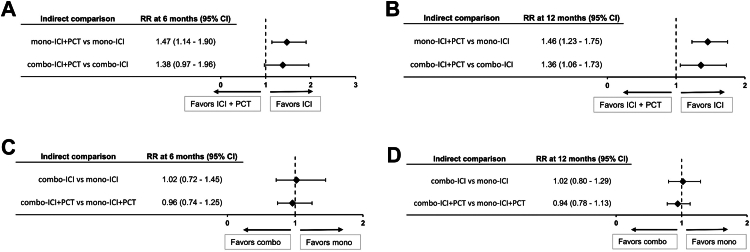
Indirect comparisons of AR risk at 6 and 12 months for PCT-containing versus PCT free regimens (A and B) and for CTLA-4 inhibitors containing versus CTLA-4 inhibitors free regimens (C and D). Abbreviations: RR, risk ratio; ICI, immune checkpoint inhibitors; PCT, platinum-based chemotherapy.

Indirect comparison of aggregated data showed a higher risk of acquired resistance both at 6 and 12 months with mono-ICI + PCT compared to mono-ICI (RR at 6 months: 1.47, 95% CI 1.14–1.90; RR at 12 months: 1.46, 95% CI 1.23–1.75) and with combo-ICI + PCT compared to combo-ICI (RR at 6 months: 1.38, 95% CI 0.97–1.96; RR at 12 months: 1.36, 95% CI 1.06–1.73) (Fig. 3A and B).

Time to event outcomes were reconstructed from the KM curves of 17 RCTs through the IPD-from-KM method. The graphical reconstruction algorithm derived IPD that resulted in HRs similar to those of the original reported curves. A side-by-side comparison of the original curves and the reconstructed curves demonstrated a close match to original KM curves on visual inspection, marginal median DoR, and comparisons of number-at-risk tables (Supplementary Table S5).

**Fig. 4:**
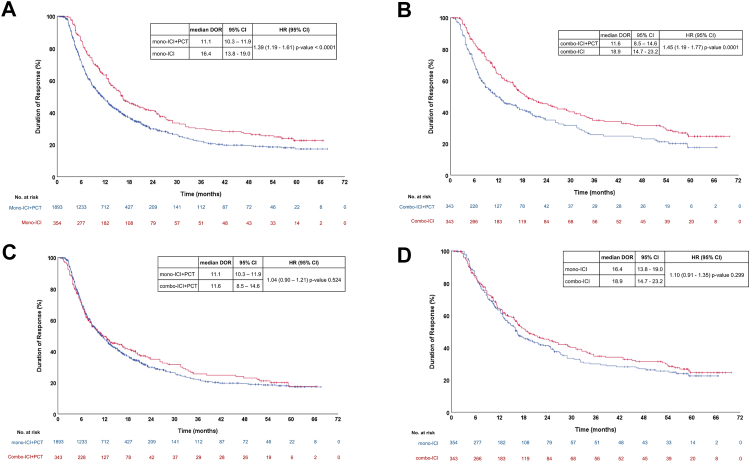
IPD reconstructed Kaplan–Meier curves comparing median duration of response between mono-ICI + PCT versus mono-ICI (A), combo-ICI + PCT versus combo-ICI (B), mono-ICI + PCT versus combo-ICI + PCT (C), mono-ICI versus combo-ICI (D). Abbreviations: DoR, duration of response; ICI, immune checkpoint inhibitors; HR, hazard ratio; PCT, platinum-based chemotherapy.

KM reconstruction showed a significantly worse median DoR with mono-ICI + PCT compared to mono-ICI [11.1 months (95% CI 10.3–11.9) versus 16.4 months (95% CI 13.8–19.0), 14 RCTs, 2247 participants, p < 0.001] and with combo-ICI + PCT compared to combo-ICI [11.6 months (95% CI 8.5–14.6) versus 18.9 months (95% CI 14.7–23.2), 6 RCTs, 686 participants p < 0.001] (Fig. 4A and B).

Censoring imbalance by reverse KM-method showed a significant difference in the likelihood of censoring between mono-ICI + PCT versus mono-ICI (HR 1.40, 95% CI 1.17–1.68, p = 0.001), and combo-ICI versus combo-ICI + PCT (HR 1.48, 95% CI 1.16–1.87, p = 0.001) (Supplementary Fig. S7A and B). Considering the possibility of a selection bias for studies in the PCT-free arms which enrolled patients with high PD-L1 expression or TMB, we conducted a sensitivity analysis excluding RCTs conducted in PD-L1 and TMB enriched populations (KEYNOTE 598, NEPTUNE, PEARL) from both the mono-ICI and combo-ICI arms. We still found a statistically significant difference in favour of the PCT-free regimens (mono-ICI + PCT versus mono-ICI: HR 1.36, 95% CI 1.06–1.73, p = 0.013; combo-ICI + PCT versus combo-ICI: HR 1.51, 95% CI 1.20–1.90, p = 0.002) (Supplementary Fig. S8).

Indirect comparison of aggregated data showed no difference in the risk of acquired resistance both at 6 and 12 months both with combo-ICI compared to mono-ICI (RR at 6 months: 1.02, 95% CI 0.72–1.45; RR at 12 months: 1.02, 95% CI 0.80–1.29) and with combo-ICI + PCT compared to mono-ICI + PCT (RR at 6 months: 0.96, 95% CI 0.74–1.25; RR at 12 months: 0.94, 95% CI 0.78–1.13) (Fig. 3C and D).

Time to event outcomes were reconstructed from the KM curves of 17 RCTs and matching with original KM curves was assessed for quality check (Supplementary Table S5). No significant difference in median DoR was observed neither with combo-ICI compared to mono-ICI [18.9 months (95% CI 14.7–23.2) versus 16.4 months (95% CI 13.8–19.0), 4 RCTs, 697 participants, p = 0.299], nor with combo-ICI + PCT compared to mono-ICI + PCT [11.6 months (95% CI 8.5–14.6) versus 11.1 months (95% CI 10.3–11.9), 14 RCTs, 2236 participants, p = 0.524] (Fig. 4C and D).

Censoring imbalance by reverse KM-method showed no significant difference in the likelihood of censoring between mono-ICI + PCT versus combo-ICI + PCT (HR 1.08, 95% CI 0.90–1.31, p 0.370) and mono-ICI compared to combo-ICI (HR 1.17, 95% CI 0.93–1.47, p 0.170) (Supplementary Fig. S7C and D).

## Discussion

In recent years, the update of many RCTs in treatment naïve metastatic NSCLC patients has shown that only <15% of patients with PD-L1 ≥ 50%^17^ and <10% of patients with any PD-L1 expression^18^^,^^19^ receiving PD-1/PD-L1 inhibitors alone or in combination with PCT are alive without progression at 5 years, being potentially cured by ICI treatment. Similarly, the 5 years progression-free survival (PFS) rate with regimens containing CTLA-4 inhibitors^20^^,^^21^ in patients unselected for PD-L1 expression was ≃10%. These data have dampened the initial enthusiasm regarding the long-lasting survival benefit of ICI in metastatic NSCLC patients, suggesting that a relatively small percentage of patients are definitively cured, and the vast majority progress and die because of primary or acquired resistance.

In the present manuscript, we conducted a systematic assessment of acquired resistance across RCTs including different ICI regimens and combinations, aiming to explore the impact of PCT and CTLA-4 inhibitors on the rate of acquired resistance and the effect on long term survival outcomes. We found that ≃15%% and ≃30% of NSCLC patients initially responding to mono-ICI, progressed within 6 or 12 months respectively. While the combination with CTLA-4 inhibitors did not appear to influence acquired resistance, indirect comparisons suggested that the addition of PCT was associated with a higher 6-months (≃35%) and 12-months (≃45%) acquired resistance rate. This 10%–15% increase in acquired resistance rate corresponded to an estimated ≃50% and ≃40% gain in the relative risk of developing acquired resistance within 6- or 12-months and to an approximately 5–7 months lower median DoR with PCT-containing compared to no PCT-regimens.

While most of the current literature is focused on the identification of mechanisms of acquired resistance in patients treated with mono or combo-ICI,^7^^,^^8^^,^^22^ evidence regarding the impact of PCT on such acquired resistance nodes is scant. Ricciuti et al. reported lower acquired genomic changes in patients with acquired resistance upon ICI + PCT compared to patients experiencing acquired resistance upon mono-ICI or combo-ICI.^22^ However, in that study only 31 patients were treated with ICI + PCT and acquired resistance included patients with progressive disease after both an initial partial response or stable disease lasting for at least 3 months, a definition which does not exactly match with the acquired resistance criteria used in the present analysis. Besides the mutational burden, PCT may also affect the immunogenicity of mutations. Indeed, neoantigen loss of truncal genomic alterations was associated with acquired resistance upon mono or combo-ICI.^23^ The effect of PCT on such clonal tumour mutations is currently unknown; however, it is likely that an increase of subclonal and less immunogenic alterations occurring with PCT may negatively affect ICI response.^24^

Regarding the effect of PCT on host immune response, although chemotherapy may help to reduce or rewire myeloid inflammation preventing primary resistance,^25^ few studies have explored the biological effects of PCT on adaptive immunity features associated with acquired resistance. Recently, Mariniello et al. reported that cisplatin and pemetrexed chemotherapy administered concomitantly to PD-1 blockade may impair effector functions of stem-like CD8 T-cells, while sequential strategy preserved their proliferative response.^26^ Similarly, Huo et al. showed that a 3-days delay between PCT and PD-1 inhibitors was associated with higher response and longer PFS compared to simultaneous PCT and ICI administration, due to the downregulation of PD-1 expression and the impairment of memory T-cells in the first 2-days after the administration of PCT.^27^ In addition, PCT may markedly reduce B-cells,^28^ which are emerging as key players for the establishment of memory response and prolonged benefit upon ICI.^29^ Finally, although chronic steroid therapy has been associated with detrimental effect on ICI survival outcome in NSCLC patients,^30^ whether their periodical administration as premedication for pemetrexed and paclitaxel chemotherapy, could impact on long lasting benefit from ICI + PCT combinations remains uncertain.

Regarding the effect of CTLA-4 inhibition on acquired resistance, even though the addition of CTLA-4 inhibitors could raise the tails of DoR KM curves in some studies (i.e., Checkmate 227 and Checkmate 9LA) compared to anti-PD-1 agents, we did not observe a clear role for CTLA-4 inhibitors in reducing acquired resistance risk and prolonging DoR in patients with NSCLC receiving a PD-1/PD-L1 blockade backbone. From a biological point of view, a potential synergism between CTLA-4 and PD-1 blockade has been reported because both checkpoints converge to downregulate CD28 coactivation signal on T-cells.^31^ However, optimal biomarkers for CTLA-4 inhibitors in NSCLC have not yet been determined and the biological effect of CTLA-4 blockade on T-regulatory cells is still a matter of debate,^32^ adding further complexity to the acquired resistance topic. Recently, mutations in *KEAP1* or *STK11* genes have been associated with potential benefit from the addition of anti-CTLA-4 agents,^33^ however, DoR stratified according to *KEAP1* and *STK11* mutations were not available from RCTs to test the impact of CTLA-4 inhibition on acquired resistance in such specific genomic subgroups.

We acknowledge that this analysis has several limitations. First, both PD-L1 expression and TMB are factors potentially associated with a longer DoR. The PD-L1 expression has been correlated with increased ORR, in particular, patients with PD-L1 expression ≥ 90% experienced a deeper response and longer PFS compared to patients with PD-L1 expression ≥ 50% but <90%.^34^^,^^35^ Higher PD-L1 expression is also more common in patients with acquired resistance compared to patients with primary resistance, in line with an upregulation of IFN-ɣ chronic inflammatory response in patients progressing upon ICI after an initial response.^8^ Most of the RCTs enrolling patients with PD-L1 ≥ 50% (i.e., Keynote 024, IMPower110, Empower Lung 01) were not eligible for the present analysis, due to the absence of DoR data. Among the 19 included RCTs, only four (CheckMate 227 part 1, CheckMate 9LA, KEYNOTE 598, PEARL) reported DoR KM curves according to PD-L1 expression. In particular, no study testing a PCT-containing regimen and only 3 PCT-free studies (CheckMate 227 part 1, KEYNOTE 598 and PEARL) published KM curves in PD-L1 ≥ 50% category. Therefore, we could not compare DoR between PCT-containing versus PCT-free regimens in high PD-L1 subpopulation and whether the level of PD-L1 expression may influence the potential negative impact of PCT on DoR upon ICI remains unclear. Similarly, higher TMB was associated with oligo-acquired resistance to ICI (<3 new or progressing lesions) in NSCLC patients^36^ suggesting that a persistently inflamed tumour microenvironment with increased number of mutations and PD-L1 expression is a common pattern in acquired resistance. Among the 19 included RCTs, only NEPTUNE reported the DoR KM curves stratified by bTMB, while MYSTIC reported the median DoR (with no curves) according to bTMB.^37^^,^^38^ Therefore, it was not possible to assess the impact of TMB level on acquired resistance upon ICI. It’s likely that higher PD-L1 expression and TMB level might be both associated with a longer DoR, as reported in Checkmate 227 (for PD-L1 1% threshold) and MYSTIC (for bTMB 20 mut/Mb cut off). However, PD-L1 expression and TMB suffer from high intrapatient spatial and temporal heterogeneity.^2^ In addition, a phase II trial of first-line single-agent pembrolizumab in NSCLC patients with PD-L1 expression <50% (including ≃30% of PD-L1 negative patients) showed a median DoR of 14.5 months with the upper CI of 24.9 months.^36^ Likewise, in the second line setting, no difference in median DoR with single agent nivolumab^39^ or atezolizumab^40^ was observed according to PD-L1 expression level. Besides PD-L1 expression and TMB, other factors such as tobacco exposure, patients’ ethnicity, presence of genomic alterations (i.e., ROS-1, RET, NTRK1 fusions, HER2, BRAF or MET mutations) usually not tested in many RCTs, may impact on the duration of response to ICI, potentially influencing the results of the current analysis.

The combined influence of these variables, together with the lack of stratified DoR data, limits the ability to robustly assess their impact on acquired resistance in the context of ICI therapy.

Moreover, while the analysis of acquired resistance was based on patients who achieved an objective response, it is important to acknowledge that the addition of PCT to ICI may reduce the risk of primary resistance to immunotherapy,^4^ thereby increasing the likelihood of achieving an objective response and enabling the assessment of the subsequent acquired resistance occurrence.

Another potential limitation concerns the heterogeneity of acquired resistance patterns. Different groups have highlighted that oligo and systemic acquired resistance are different prognostic categories, and are associated with specific clinical features and translational biomarkers.^9^^,^^41^ Similarly, baseline tumour burden^42^ and metastatic involvement of specific anatomical sites, such as liver^43^ or bone,^44^ can further jeopardize results from the present study. Unfortunately, most included RCTs did not provide information on type of acquired resistance and DoR data were not stratified according to baseline tumour burden and metastatic sites.

Finally, although methodological precautions (through quality checks) have been applied to ensure that the derived KM and number-at-risks are similar to the ones reported in the original RCTs, we acknowledge that differences in censoring could be inaccurate and patient level covariates are impossible to predict, given the lack of access to participants individual level data. In this regard, we also found a significant censoring imbalance in the combo-ICI versus mono-ICI and in the combo-ICI–PCT versus mono-ICI-CT favouring combo-ICI regimens. For this reason, we advise clinicians to refer to future updates on published data especially for combo-ICI results. Likewise, the indirect comparison between different treatment regimens cannot replace head-to-head comparative studies assessing the impact of PCT or CTLA-4 inhibitors on DoR.

Despite these limitations, the present analysis suggests a more careful patients’ selection at baseline by raising the possibility of a negative impact of PCT addition on acquired resistance upon ICI. Indeed, although PCT may help to prevent primary resistance, especially in patients with high disease burden and poor prognostic factors, PCT-free regimens (mono-ICI or combo-ICI) could achieve better long-term results in patients with low risk of early mortality, by potentially delaying the emergence of acquired resistance. By evaluating the acquired resistance rates across different treatment types, this study lies the groundwork for a more comprehensive characterization of primary and acquired resistance features and for their integration with existing biomarkers (i.e., PD-L1 expression), ultimately supporting improved patients’ selection and informing clinicians’ therapeutic decisions.

## Contributors

**Sara Oresti**: investigation and writing original draft; **Fabio Salomone**: methodology, formal analysis and data curation; **Antonio Nuccio**: formal analysis and data curation; **Francesca Rita Ogliari**: visualization and supervision; **Silvia Teresa Riva**: supervision; **Ludovica Mollica**: supervision; **Alessandra Bulotta**: supervision; **Maria Grazia Viganò**: writing original draft; **Francesco Maria Venanzi**: methodology and resources; **Francesco Passaretti**: methodology and resources; **Ludovica Papotto**: investigation; **Anna Di Lello**: data curation; **Michele Ferrara**: formal analysis; **Giorgia Foggetti**: writing review and editing; **Giuseppe Damiano**: supervision; **Alberto Servetto**: methodology; Raffaele Califano: writing review and editing; Massimo Di Maio: writing review and editing; **Biagio Ricciuti**: data curation; **Alessio Cortellini**: investigation; **Martin Reck**: writing review and editing; **Michela Cinquini**: methodology and software; **Valter Torri**: methodology and software; **Giuseppe Viscardi**: data curation, writing original draft; **Roberto Ferrara**: conceptualization and writing original draft.

All authors had full access to all the data in the study and had final responsibility for the decision to submit for publication.

## Data sharing statement

This review is based on publicly available, or fee-based accessible, peer-reviewed manuscripts. Moreover, the authors’ search strategies for PubMed and EMBASE have been made available as part of this publication.

## Declaration of interests

Sara Oresti reports Speaker’s fee from AstraZeneca and Roche; Support for attending meetings and/or travel from Jannses, Johnson & Johnson and Takeda. Francesca Rita Ogliari reports Speaker’s fee from Roche, Sanofi, Amgen; Support for attending meetings and/or travel from Amgen, Johnson & Johnson. Raffaele Califano reports grants or contracts from Merck Sharp & Dohme, Roche, PharmaMar, GSK, Janssen, AstraZeneca, Taiho, ArriVent and OSE Immunotherapeutics; consulting fees from Pfizer, Merck Sharp & Dohme, PharmaMar, ArriVent, Roche, Bristol Myers Squibb, AstraZeneca, Biontech, Janssen, GSK and Takeda; payment or honoraria from Beigene, Regeneron, AstraZeneca, Janssen, GSK, Takeda; support for attending meetings and/or travel from Janssen, Takeda; participation on a data safety monitoring board or advisory board for PharmaMar, Janssen, Astrazeneca, Arrivent; leadership or fiduciary role for ESMO Educational Publication Working Group; stock or stock options with Supportive Care UK and LOC at the Christie Private Care. Bulotta Alessandra reports honoraria for a role as consultant and advisory board for Roche, BMS, AstraZeneca and speaker fees for BMS, MSD, AstraZeneca, Ely Lilly. Biagio Ricciuti reports speaker fees from Targeted Oncology, consultancy or participation to advisory board from Regeneron, AstraZeneca, Amgen, Guidepoint Inc. Alessio Cortellini reports grants for consultancies/advisory boards from MSD, BMS, IQVIA, AstraZeneca, REGENERON, Amgen, Daiichi-Sankyo, Access Infinity, Ardelis Health, Alpha Sight, Guidepoint, Roche and Alira Health; speaker fees from AstraZeneca, Pierre-Fabre, MSD, Sanofi/REGENERON; payment for writing/editorial activity from BMS, MSD, Roche; travel support from Sanofi/REGENERON, MSD. Martin Reck reports speaker fees from Amgen, AstraZeneca, Beigene, Boehringer Ingelheim, Bristol Myers Squibb, Daiichi Sankyo, GSK, Mirati, Merck, MSD, Lily, Novartis, Pfizer, Sanofi, Roche, and Regeneron; consultancy or participation to advisory board from Daiichi Sankyo and Sanofi. Alberto Servetto reports honoraria from Eli Lilly, MSD, and Janssen and travel support from Bristol-Myers Squibb and AstraZeneca. Massimo Di Maio reports honoraria from Pfizer, Takeda, AstraZeneca, Janssen, Eisai, Novartis, Roche, Astellas Pharma, MSD Oncology, Boehringer Ingelheim, Viatris, Ipsen; consultancy or participation to advisory boards and direct research funding from AstraZeneca, Pfizer, Takeda, Janssen, Eisai, Novartis, Roche, MSD, Viatris, Ipsen; Research Funding from Tesaro/GlaxoSmithKline. Giuseppe Viscardi reports travel support from AstraZeneca, MSD, Novartis, Sanofi; honoraria for advisory board from Amgen, MSD, Novartis. Ferrara Roberto reports honoraria for advisory board from MSD, Beigene, AstraZeneca, BMS, Sanofi, Johnson & Johnson.
