# Supplementary material for: Impact of platinum-based chemotherapy and CTLA-4 inhibition on acquired resistance to first-line anti-PD-1/PD-L1 agents in non-small cell lung cancer: a systematic review and reconstructed individual patient data analysis
Source: eClinicalMedicine. 2025 Sep 2;88:103482. doi: 10.1016/j.eclinm.2025.103482 (PMC12441679; doi:10.1016/j.eclinm.2025.103482)

**Supplementary content**

**Supplementary Table 1.** Search strategy

**Supplementary Table 2.** Clinical characteristics of patients included in the studies. Abbreviations: Cnt control; Exp experimental; NA not available. * These groups include “rest of the world”. not only Caucasian population.

**Supplementary Table 3.** Causes of bias in included studies.

**Supplementary Table 4.** 6- and 12-months AR rates for each trial

**Supplementary Table 5.** Comparison between results from the individual patient data Kaplan Meier reconstruction and the original published trials.

**Supplementary Figure 1.** Risk of bias summary percentages across all the included studies.

**Supplementary Figure 2.** Risk of bias summary for each included study

**Supplementary Figure 3.** Publication bias assessment by funnel plot for mono-ICI + PCT versus PCT

**Supplementary Figure 4.** Acquired resistance rates at 6 (A) and 12 (B) months according to treatment regimens (17 RCTs).

**Supplementary Figure 5.** Comparison of AR risk at 6 months between mono-ICI +PCT versus PCT (A). combo-ICI + PCT versus PCT (B). mono-ICI versus PCT (C). combo-ICI versus PCT (D).

**Supplementary Figure 6.** Comparison of AR risk at 12 months between mono-ICI +PCT versus PCT (A). combo-ICI + PCT versus PCT (B). mono-ICI versus PCT (C). combo-ICI versus PCT (D)

**Supplementary Figure 7.** Censoring imbalance assessment by reverse Kaplan Meier method for the mono-ICI+PCT versus mono-ICI (A). combo-ICI+PCT versus combo-ICI (B). mono-ICI+PCT versus combo-ICI+PCT (C). mono-ICI versus combo-ICI (D).

**Supplementary Figure 8.** Sensitivity analysis of mono-ICI+PCT versus mono-ICI (A). combo-ICI+PCT versus combo-ICI (B) eliminating RCTs in PD-L1 and TMB selected populations.

**Supplementary Table 1.** Search strategy

**MEDLINE (Pubmed) (1946 to January 31. 2025)**

| #1 | (((((((((((("Carcinoma. Non-Small-Cell Lung"[Mesh]) OR ("lung cancer"[Title/Abstract] OR "lung neoplasm*"[Title/Abstract] OR "lung tumo*"[Title/Abstract] OR "Non-small-cell lung cancer"[Title/Abstract] OR "Non small cell lung cancer"[Title/Abstract])) OR NSCLC[Title/Abstract] OR "Non-Small-Cell Lung cancer"[Title/Abstract])))))))))))) OR (Nonsquamous[Title/Abstract] OR Non-squamous[Title/Abstract] OR Non squamous[Title/Abstract] OR Squamous[Title/Abstract])) AND ((advance*[Title/Abstract] OR metastatic*[Title/Abstract] OR "locally advance*"[Title/Abstract]) OR (("Neoplasm Staging"[Mesh]))) | 133.966 |
| --- | --- | --- |
| #2 | ((((((("Nivolumab"[Mesh] OR "durvalumab" [Supplementary Concept] OR "pembrolizumab" [Supplementary Concept] OR "cemiplimab" [Supplementary Concept] "camrelizumab" [Supplementary Concept]) OR Toripalimab[Title/Abstract] OR "atezolizumab" [Supplementary Concept] OR carrelizumab[Title/Abstract] OR Sugemalimab[Title/Abstract] OR (Nivolumab[Title/Abstract] OR Opdivo[Title/Abstract] OR Durvalumab[Title/Abstract] OR Imfinzi[Title/Abstract] OR Pembrolizumab[Title/Abstract] OR Keytruda[Title/Abstract] OR Cemiplimab[Title/Abstract] OR Camrelizumab[Title/Abstract] OR atezolizumab [Supplementary Concept] OR Atezolizumab[Title/Abstract] OR Tecentriq[Title/Abstract]) AND ("Ipilimumab"[Mesh]) OR "tremelimumab" [Supplementary Concept]) OR Ipilimumab[Title/Abstract] OR Yervoy[Title/Abstract] OR Tremelimumab[Title/Abstract] OR ticilimumab[Title/Abstract] OR Pemetrexed[Title/Abstract]))) OR (Carboplatin[Title/Abstract] OR Paclitaxel[Title/Abstract] OR Taxol[Title/Abstract] OR nab-paclitaxel[Title/Abstract]))))) AND ((chemotherap*[Title/Abstract]))) OR ((Toripalimab[Title/Abstract] OR Cemiplimab[Title/Abstract]) AND (chemotherap*[Title/Abstract)) | 33.149 |
| #4 | (((((((("Meta-Analysis as Topic"[Mesh]) OR "Meta-Analysis"[Publication Type]) OR "meta-analysis[Title] OR systematic review"[Title] OR "meta analy*"[Title] OR "metaanaly*"[Title] OR "Meta-Analysis"[Title])) OR ("systematic literature review"[Title])) OR ("systematic review"[Title/Abstract])) OR (((("Randomized Controlled Trial"[Publication Type]) OR "Randomized Controlled Trials as Topic"[Mesh]) OR ("Clinical Trial" [Publication Type])) OR (randomized[Title/Abstract] OR randomised[Title/Abstract] OR randomly[Title/Abstract])))) | 2.155.345 |
| #5 | #1 AND #2 AND #3 AND #4 | **2.219** |

**Embase (1974 to January 31. 2025)**

| #1 | 'lung tumor'/exp/mj OR 'non small cell lung cancer'/exp/mj | 166.468 |
| --- | --- | --- |
| #2 | 'lung cancer' OR 'lung neoplasm*' OR 'lung tumo*' OR 'non-small-cell lung cancer' OR 'non small cell lung cancer' OR nsclc OR 'non-small-cell lung cancer':ti.ab | 537.378 |
| #3 | #1 OR #2 | 550.379 |
| #4 | advance* OR metastatic* OR 'locally advance*':ti.ab | 2.789.369 |
| #5 | #3 AND #4 | 140.626 |
| #6 | 'nivolumab'/exp/mj OR OR 'durvalumab'/exp/mj OR 'pembrolizumab'/exp/mj OR 'camrelizumab'/exp/mj OR 'atezolizumab'/exp/mj | 30.340 |
| #7 | nivolumab OR opdivo OR durvalumab OR imfinzi OR pembrolizumab OR keytruda OR cemiplimab OR camrelizumab OR carrelizumab OR atezolizumab OR tecentriq OR Sugemalimab:ti.ab | 86.478 |
| #8 | 'ipilimumab'/exp/mj OR 'pemetrexed'/exp/mj OR 'carboplatin'/exp/mj OR 'paclitaxel'/exp/mj | 85.558 |
| #9 | ipilimumab OR yervoy OR OR tremelimumab OR ticilimumab OR Pemetrexed OR Carboplati] OR Paclitaxel OR Taxol OR nab-paclitaxel:ti.ab | 242.407 |
| #10 | (#6 OR #7) AND (#8 OR #9) | 235.209 |
| #11 | chemotherap* | 1.210.736 |
| #12 | #10 AND #11 | 17.748 |
| #13 | #5 AND #12 | 4.539 |
| #14 | toripalimab OR cemiplimab:ti.ab | 3.420 |
| #15 | #11 AND #14 | 1.687 |
| #16 | #5 AND #15 | 391 |
| #17 | #13 OR #16 AND OR [controlled clinical trial]/lim OR [randomized controlled trial]/lim) | 684 |

**Cochrane Central Register of Controlled Trials (CENTRAL; 2024. Issue 5) in the Cochrane Library (searched January 31. 2025)**

| #1 | MeSH descriptor: [Lung Neoplasms] explode all trees | 12.344 |
| --- | --- | --- |
| #2 | MeSH descriptor: [Carcinoma. Non-Small-Cell Lung] explode all trees | 7.098 |
| #3 | #1 OR #2 | 12. 344 |
| #4 | (nivolumab OR opdivo OR ipilimumab OR yervoy OR durvalumab OR imfinzi OR tremelimumab OR ticilimumab OR pembrolizumab OR keytruda OR cemiplimab OR camrelizumab OR carrelizumab OR atezolizumab OR tecentriq):ti.ab.kw | 10.551 |
| #5 | (chemotherapy):ti.ab.kw | 100.517 |
| #6 | #3 AND #4 AND #5 | 657 |

**Supplementary Table 2.** Clinical characteristics of patients included in the studies. Abbreviations: Cnt control; Exp experimental; NA not available. *These groups include “rest of the world”. not only Caucasian population.

| Trial Name | Genomic alterations tested | Smokers Exp Arm, n (%) | Smokers Cnt Arm, n (%) | Caucasian Exp Arm, n (%) | Caucasian Cnt Arm, n (%) | Asian Exp Arm, n (%) | Asian Cnt Arm, n (%) | Screening failure rate n (%) | Stratification Factors |
| --- | --- | --- | --- | --- | --- | --- | --- | --- | --- |
| CameL | EGFR/ALK | 127/205 (62.0) | 130/207 (62.8) | 0 | 0 | 205/205 (100) | 207/207 (100) | 426/845 (50.4) | Sex; Smoking history |
| CameLSQ | EGFR/ALK | 171/193 (88.6) | 173/196 (88.2) | 0 | 0 | 193/193 (100) | 196/196 (100) | 216/606 (35.6) | Smoking history; Brain or liver metastasis; Sex |
| CHOICE-01 | EGFR/ALK | 213/309 (68.9) | 107/156 (68.7) | 0 | 0 | 309/309 (100) | 156/156 (100) | 370/835 (44.3) | PD-L1 levels (TC<1% or ≥1%); Histology; Smoking status |
| EmpowerLUNG3 | EGFR/ALK/ROS1 | 269/312 (86.2) | 130/154 (84.4) | 270/312 (86.5) | 138/154 (89.6) | 42/312 (13.5) | 16/154 (10.4) | 438/904 (48.4) | Histology; PD-L1 levels (<1%. 1%–49%. or ≥50%) |
| GEMSTONE302 | EGFR/ALK/ROS1/RET | 232/320 (72.5) | 119/159 (74.8) | 0 | 0 | 320/320 (100) | 159/159 (100) | 367/846 (43.3) | ECOG PS; PD-L1 levels (TPS <1% or ≥1%); Histology |
| CheckMate227 | EGFR/ALK | 497/583 (85.2) | 499/583 (85.6) | NA | NA | NA | NA | 1137/2876 (39.5) | Histology |
| CheckMate227 part 2 | EGFR/ALK | 316/377 (83.8) | 299/378 (79.1) | 199/377 (52.7) | 181/378 (47.8) | 89/377 (23.6) | 92/378 (24.3) | 469/1224 (38.3) | Histology; Sex; PD-L1 levels (<1% or ≥1%) |
| IMpower130 | EGFR/ALK | 419/483 (86.7) | 220/240 (91.6) | 428/483 (88.6) | 222/240 (92.5) | 14/483 (2.8) | 3/240 (1.2) | 523/1247 (41.9) | Sex; Liver metastasis; PD-L1 levels (TC3 and any IC or TC0/1/2 and IC2/3 vs TC0/1/2 and IC0/1) |
| KEYNOTE021 | EGFR/ALK | 45/60 (75.0) | 54/63 (85.7) | 49/60 (81.6) | 58/63 (92.0) | 5/60 (8.3) | 5/63 (7.9) | 96/219 (43.8) | PD-L1 levels (TPS <1% or ≥1%) |
| KEYNOTE042 | EGFR/ALK | 495/637 (77.7) | 497/637 (78.0) | 149/637 (23.4) | 137/637 (21.5) | 185/637 (29.0) | 185/637 (29.0) | 2154/3428 (62.8) | Region; ECOG PS; Histology; PD-L1 levels (TPS ≥50% or 1–49%) |
| KEYNOTE189 | EGFR/ALK | 362/410 (88.3) | 181/206 (87.8) | 354/410 (86.3) | 177/206 (85.9) | 4/410 (1.0) | 6/206 (2.9) | 349/965 (36.1) | PD-L1 levels (TPS ≥1% or <1%); Platinum compound; Smoking status |
| KEYNOTE407 | NONE | 256/278 (92.1) | 262/281 (93.2) | 224/278 (80.6) * | 229/281 (81.5) * | 54/278 (19.4) | 52/281 (18.5) | 218/779 (27.9) | PD-L1 levels (TPS ≥1% or <1%); Taxane compound: Region |
| KEYNOTE598 | EGFR/ALK | 255/284 (89.8) | 259/284 (91.2) | 252/284 (88.7) * | 253/284 (89.1) * | 32/284 (11.3) | 31/284 (10.9) | 1861/2429 (76.6) | ECOG PS; Region; Histology |
| MYSTIC (durvalumab) | EGFR/ALK | 139/163 (85.3) | 141/162 (87.0) | 101/163 (61.9) | 113/162 (69.8) | 59/163 (36.2) | 47/162 (29.0) | 773/1891 (40.8) | PD-L1 levels (TC ≥25% vs <25%); Histology |
| MYSTIC (durvalumab + tremelimumab) | EGFR/ALK | 138/163 (84.7) | 141/162 (87.0) | 111/163 (68.1) | 113/162 (69.8) | 50/163 (30.7) | 47/162 (29.0) | 773/1891 (40.8) | PD-L1 levels (TC ≥25% vs <25%); Histology |
| NEPTUNE | EGFR/ALK | 338/410 (82.4) | 339/413 (82.1) | 307/410 (74.9) | 289/413 (70.0) | 86/410 (21.0) | 99/413 (24.0) | 527/1350 (39.0) | PD-L1 levels (TC ≥25% vs <25%); Histology; Smoking status |
| NIPPON | EGFR/ALK/ROS1/BRAF/MET/RET/NTRK | 130/147 (88.4) | 131/148 (89.1) | 0 | 0 | 147/147 (100) | 148/148 (100) | NA | Institution; Clinical stage; Sex; Histology; PD-L1 levels (TPS <1% or 1–49% or ≥50% or unknown) |
| PEARL | EGFR/ALK | 264/335 (78.8) | 261/334 (78.1) | 73/335 (21.8) | 61/334 (18.3) | 262/335 (78.2) | 273/334 (81.7) | 2406/3075 (78.2) | PD-L1 levels (TC 25%–49% or ≥50%) |
| POSEIDON (tremelimumab + durvalumab) | EGFR/ALK | 279/338 (82.5) | 257/337 (76.2) | 205/338 (60.6) | 179/337 (53.1) | 99/338 (29.3) | 128/337 (38.9) | 794/1807 (43.9) | PD-L1 levels (TC ≥50% vs <50%); Histology; Disease stage |
| POSEIDON (durvalumab) | EGFR/ALK | 254/338 (75.1) | 257/337 (76.2) | 182/338 (53.8) | 179/337 (53.1) | 123/338 (36.4) | 128/337 (38.9) | 794/1807 (43.9) | PD-L1 levels (TC ≥50% vs <50%); Histology; Disease stage |

**Supplementary Table 3.** Causes of bias in include studies.

| **Trial** | **Type of bias** | **Cause** |
| --- | --- | --- |
| Camel | Performance bias | Open-label design may affect duration of response |
| CheckMate 9LA | Performance bias | Open-label design may affect duration of response |
| IMpower130 | Performance bias  Detection bias | Open-label design may affect duration of response  Investigator-assessed progression-free survival may affect duration of response |
| KEYNOTE021 | Performance bias | Open-label design may affect duration of response |
| KEYNOTE042 | Performance bias | Open-label design may affect duration of response |
| MYSTIC | Performance bias | Open-label design may affect duration of response |
| NEPTUNE | Performance bias  Detection bias  Attrition bias | Open-label design may affect duration of response  Investigator-assessed progression-free survival may affect duration of response  Amended study design. with a resultant small primary analysis population |
| NIPPON | Performance bias  Detection bias  Reporting bias | Open-label design may affect duration of response  Investigator-assessed progression-free survival may affect duration of response  Early termination of accrual. planned interim analyses not performed |
| PEARL | Performance bias  Detection bias  Reporting bias | Open-label design may affect duration of response  Investigator-assessed progression-free survival may affect duration of response  Primary objective modified during the trial to include an additional co-primary population |
| POSEIDON | Performance bias | Open-label design may affect duration of response |

**Supplementary Table 4.** 6- and 12-months AR rates for each trial

|  | **6-months AR; n (%; 95% CI)** | **12-months AR; n (%; 95% CI)** |
| --- | --- | --- |
| **CameL** | 25/113 (22.1; 14.8 – 30.9) | 48/113 (42.4; 33.2 -52.1) |
| **CameLSQ** | 33/125 (26.4; 18.9 – 35.0) | 53/125 (42.4; 33.6 – 51.5) |
| **CheckMate227** | 24/67 (36.3; 24.4 – 48.4) | 42/67 (62.6; 50.0 – 74.2) |
| **CheckMate227** | 14/109 (12.8; 7.2 – 20.6) | 34/109 (31.1; 22.6 – 40.7) |
| **CheckMate227** | 35/195 (17.9; 12.8 – 24.0) | 60/195 (30.7; 24.3 – 37.7) |
| **CheckMate227 part B** | 51/194 (26.2; 20.2 – 33.0) | 96/194 (49.4; 42.2 – 56.7) |
| **CheckMate9LA** | 35/137 (25.5; 18.4 – 33.7) | 65/137 (47.4; 38.8 – 56.1) |
| **CHOICE-01** | 60/203 (29.5; 23.3 – 36.3) | 97/203 (47.7; 40.7 – 54.8) |
| **EmpowerLUNG03** | 17/136 (12.5; 7.4 – 19.2) | 48/136 (35.2; 27.3 – 43.9) |
| **GEMSTONE302** | 64/203 (31.5; 25.2 – 38.4) | 105/203 (51.7; 44.6 – 58.7) |
| **IMpower130** | 64/220 (29.0; 23.1 – 35.5) | 112/220 (50.9; 44.1 – 57.6) |
| **KEYNOTE021** | 3/35 (8.5; 1.8 -23.0) | 5/35 (14.2; 4.8 – 30.2) |
| **KEYNOTE042** | 21/174 (12.0; 7.6 – 17.8) | 57/174 (32.7; 25.8 – 40.2) |
| **KEYNOTE189** | 28/198 (14.1; 9.6 – 19.7) | 86/198 (43.4; 36.4 – 50.6) |
| **KEYNOTE407** | 56/173 (32.3; 25.4 – 39.8) | 95/173 (54.9; 47.1 – 62.4) |
| **MYSTIC** | 18/66 (27.2; 17.0 – 39.6) | 23/66 (34.8; 23.5 – 47.5) |
| **MYSTIC** | 16/65 (24.6; 14.7 – 36.8) | 24/65 (36.9; 25.2 – 49.8) |
| **NEPTUNE** | 2/19 (10.5; 1.3 – 33.1) | 8/19 (42.1; 20.2 – 66.5) |
| **PEARL** | 24/116 (20.6; 13.7 – 29.2) | 45/116 (38.7; 29.8 – 48.2) |
| **POSEIDON** | 53/137 (38.6; 30.4 – 47.3) | 75/137 (54.7; 46.0 – 63.2) |
| **POSEIDON** | 41/130 (31.5; 23.6 – 40.2) | 61/130 (46.9; 38.1 – 55.8) |

**Supplementary Table 5.** Comparison between results from the individual patient data Kaplan Meier reconstruction and the original published trials.

| **Original Article** | **DOI** | **Reconstructed Kaplan-Meier** |
| --- | --- | --- |
| CameL | 10.1136/jitc-2024-009240 | 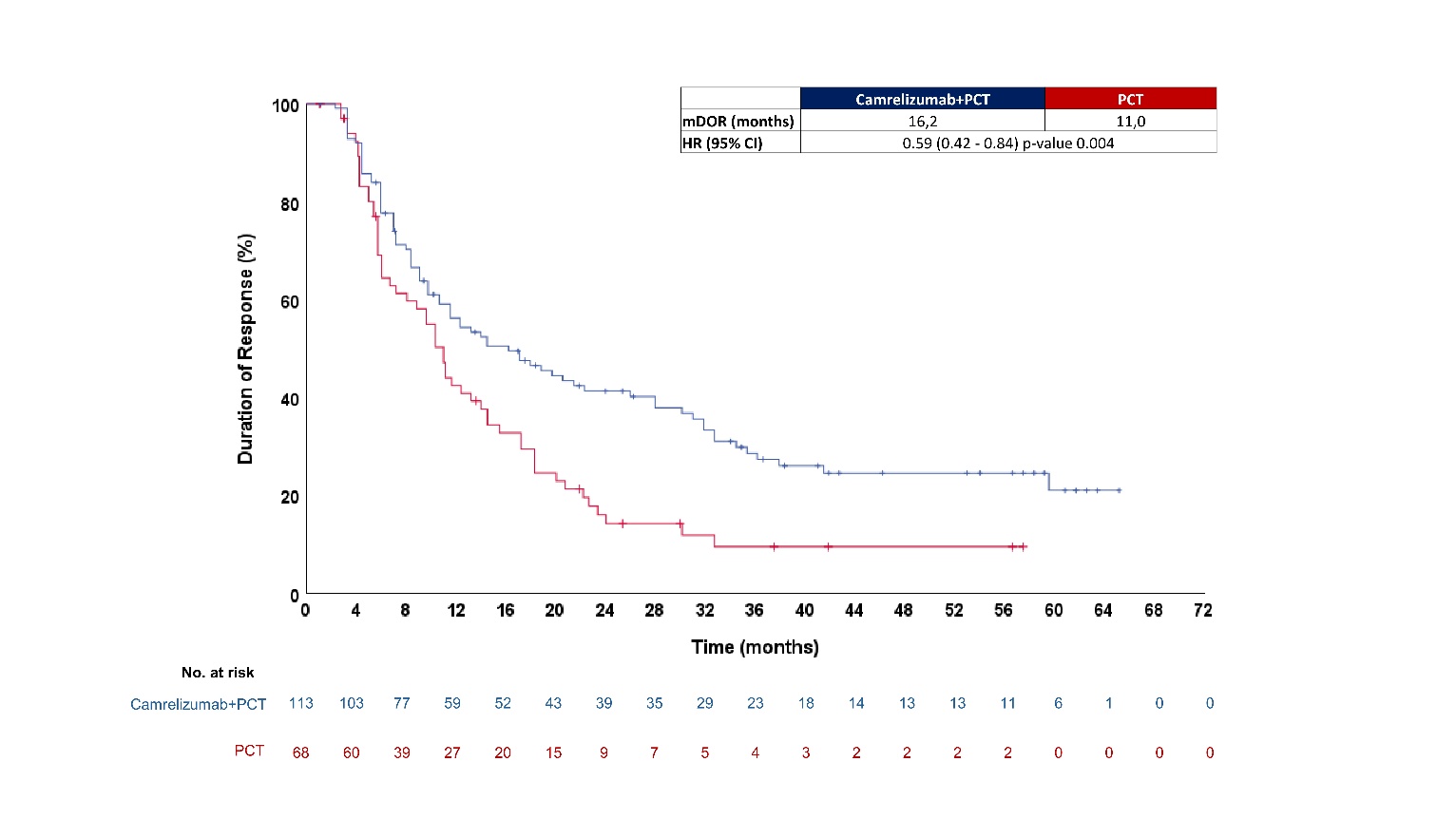 |
| CameLSQ | 10.1016/j.jtho.2021.11.018 | 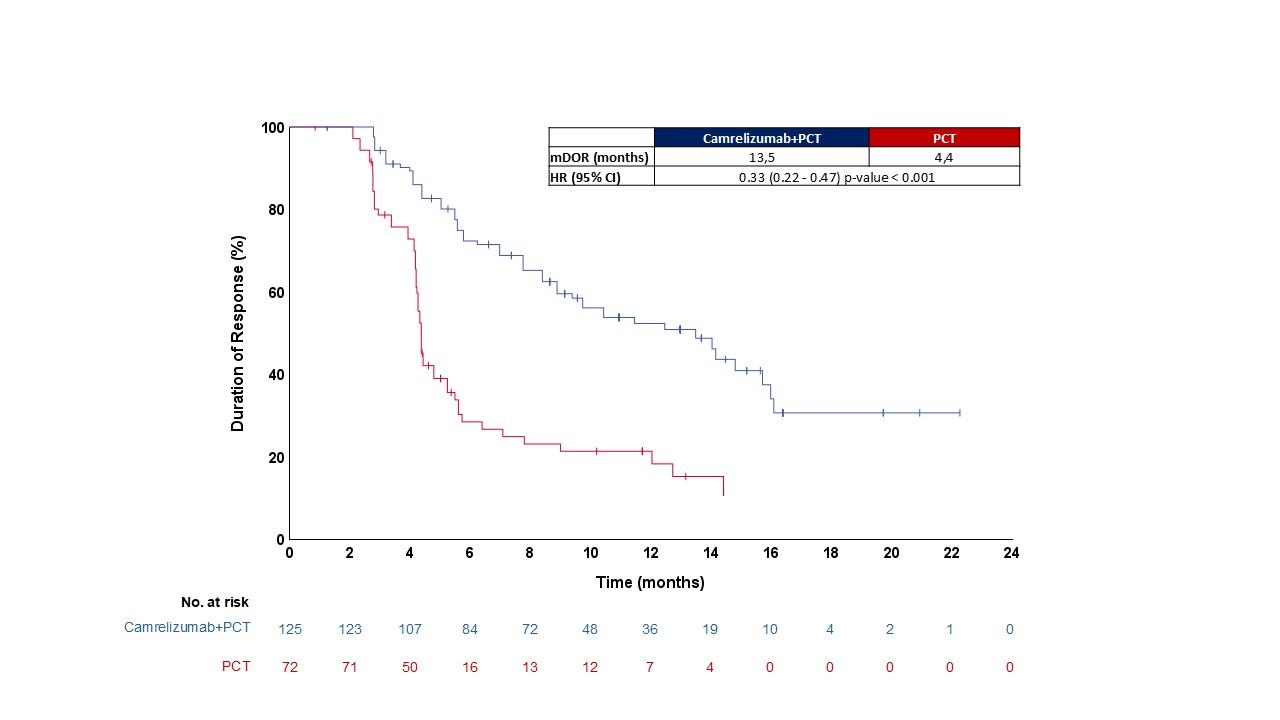 |
| CheckMate9LA | 10.1016/j.ejca.2024.114296 | 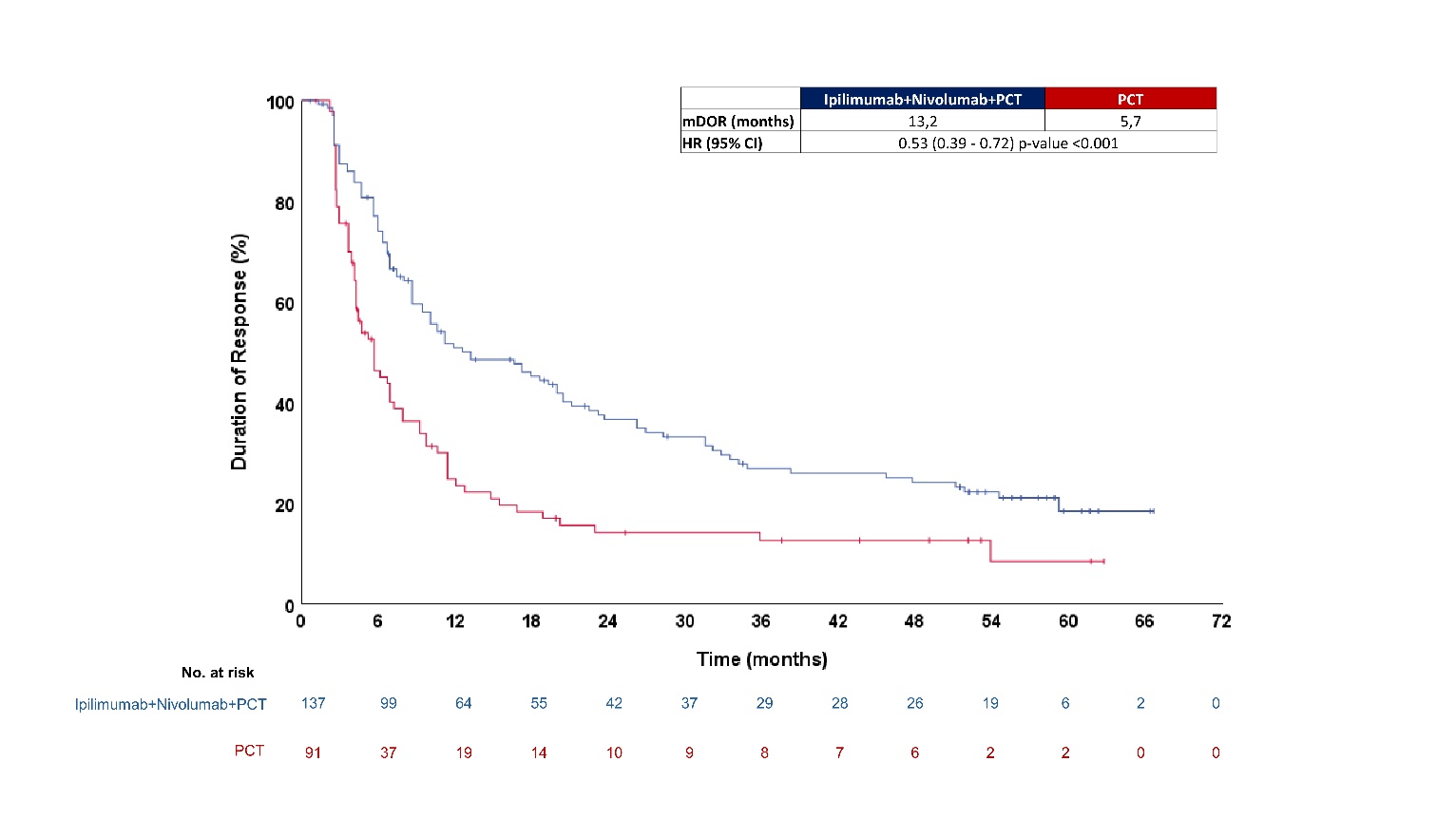 |
| CheckMate227  (PD-L1≥1%) | 10.1200/JCO.22.01503 | 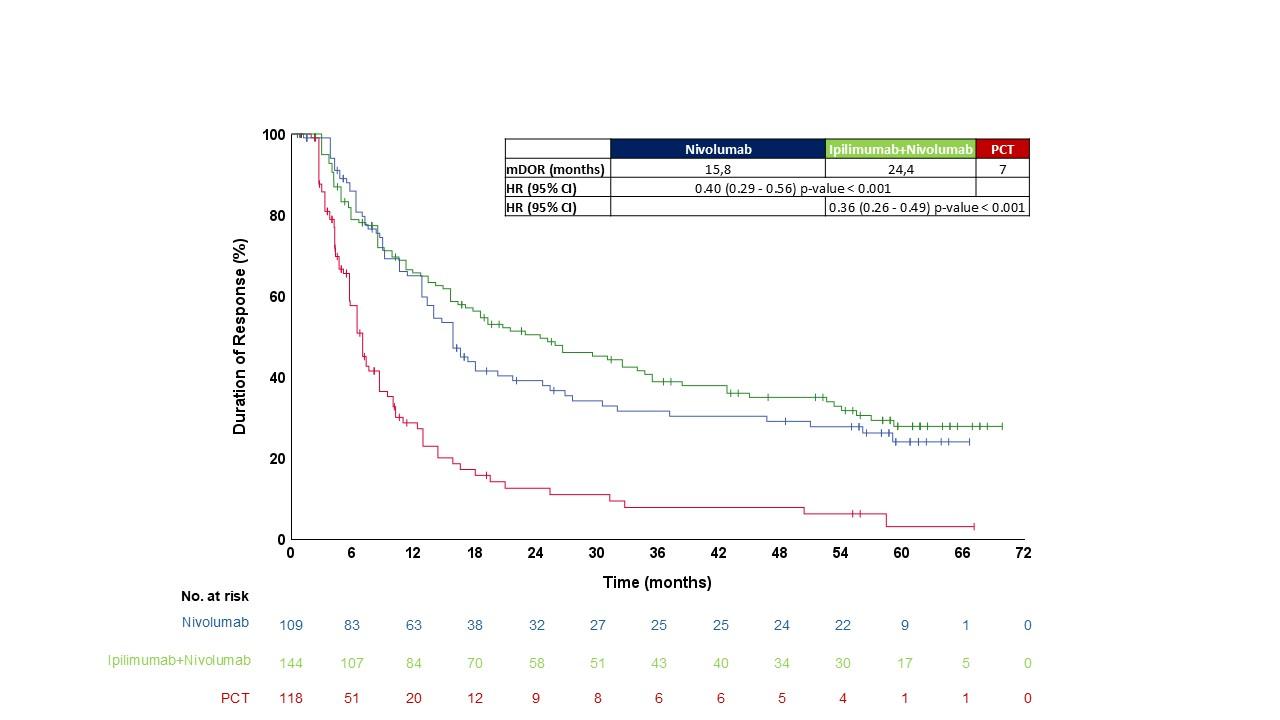 |
| CheckMate227  (PD-L1<1%) | 10.1200/JCO.22.01503 | 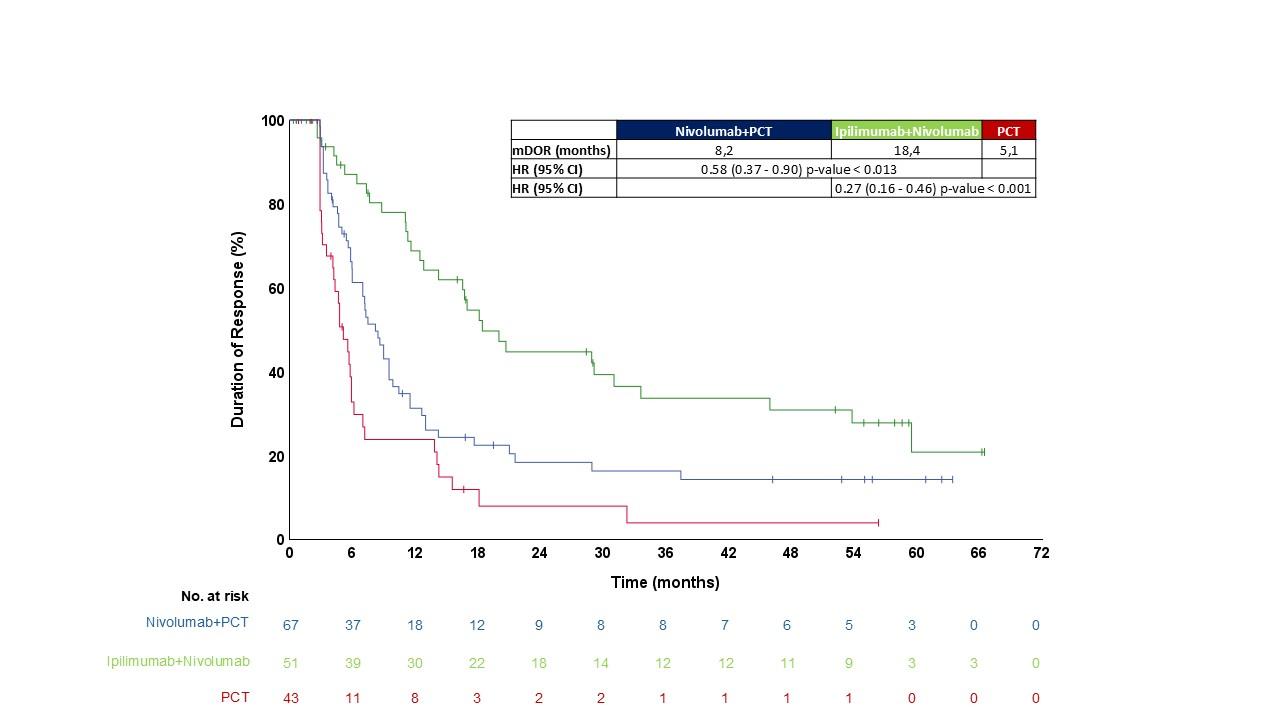 |
| CheckMate227 part 2 | 10.1016/j.esmoop.2023.102065 | 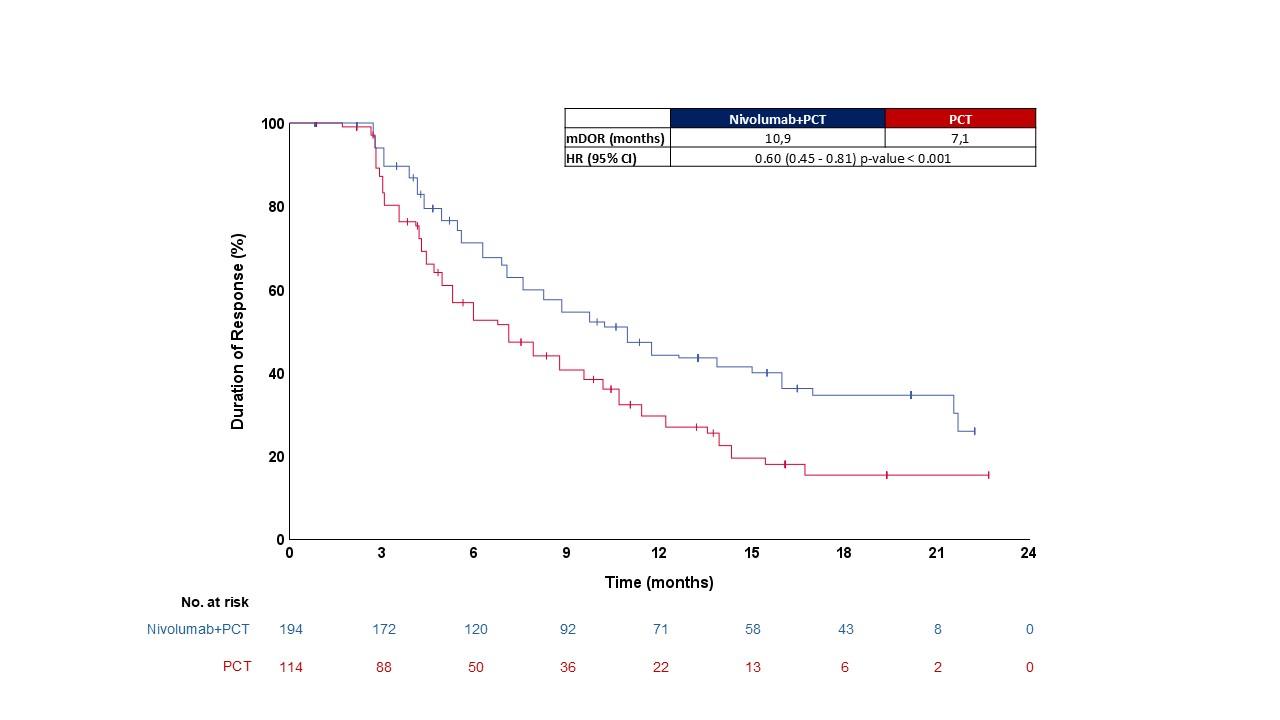 |
| CHOICE01 | 10.1200/JCO.22.00727 | 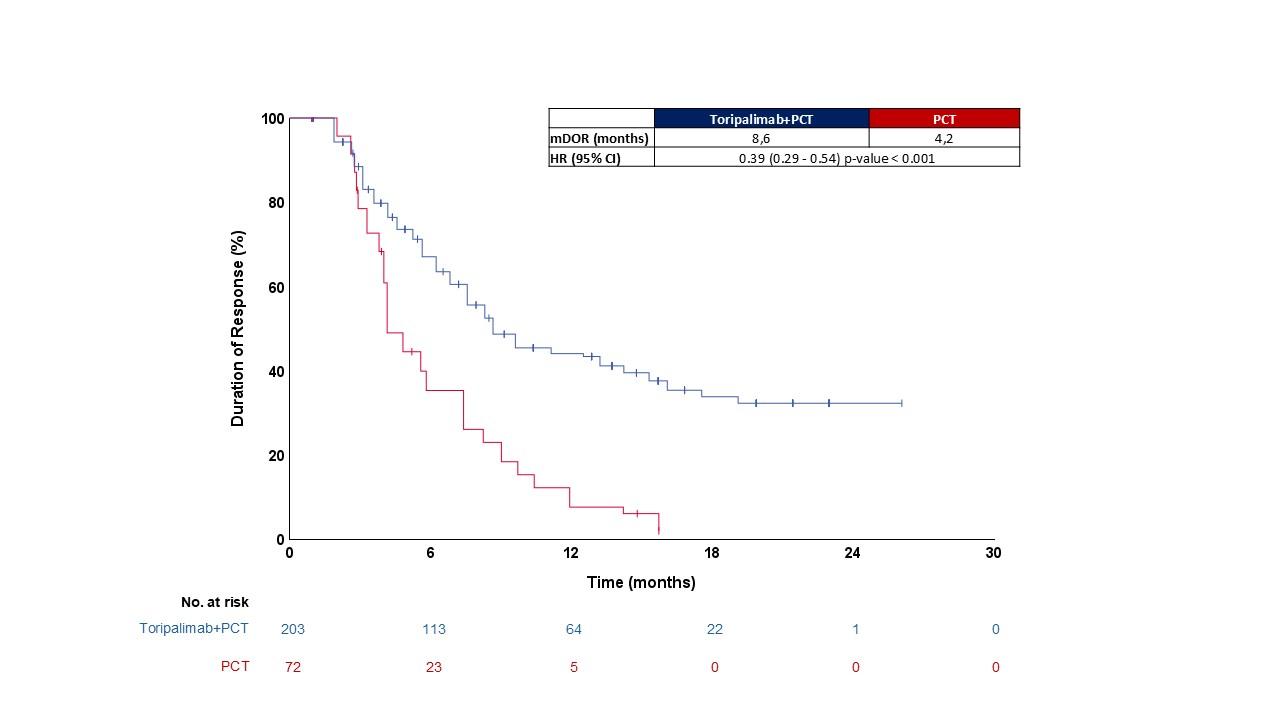 |
| EmpowerLUNG03 | 10.1016/j.jtho.2023.03.008 | 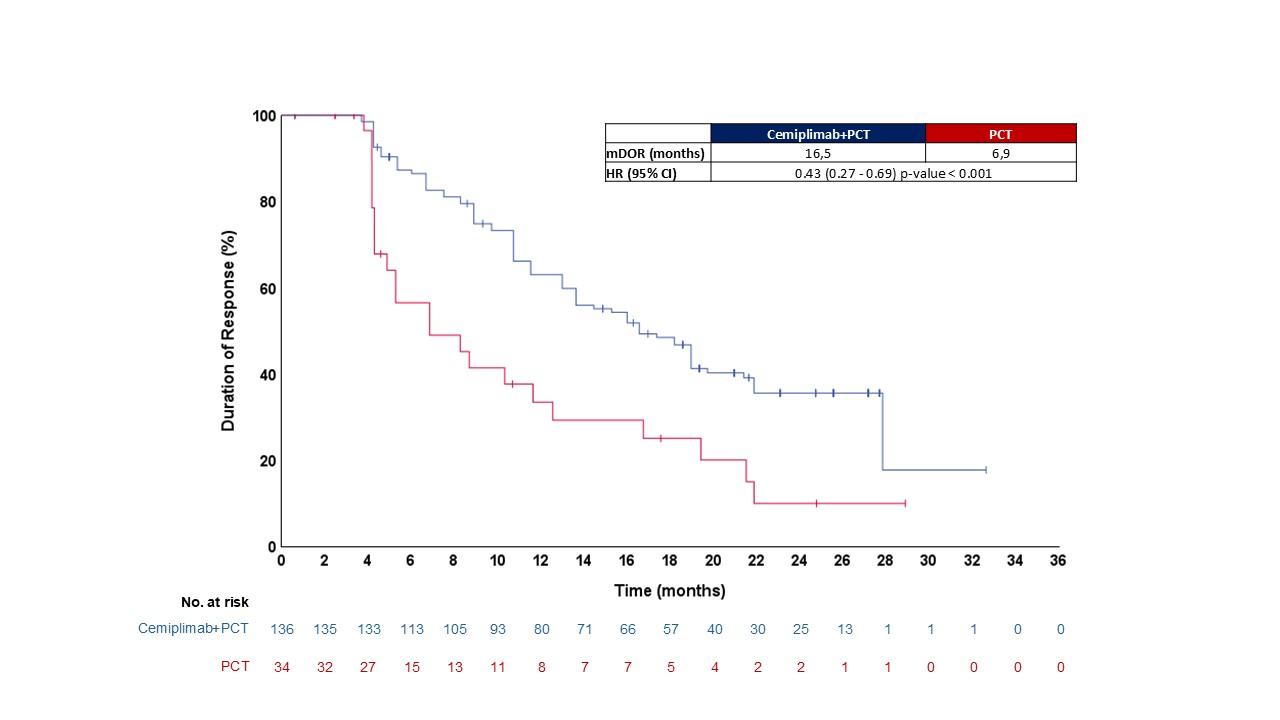 |
| GEMSTONE302 | 10.1038/s43018-023-00578-z | 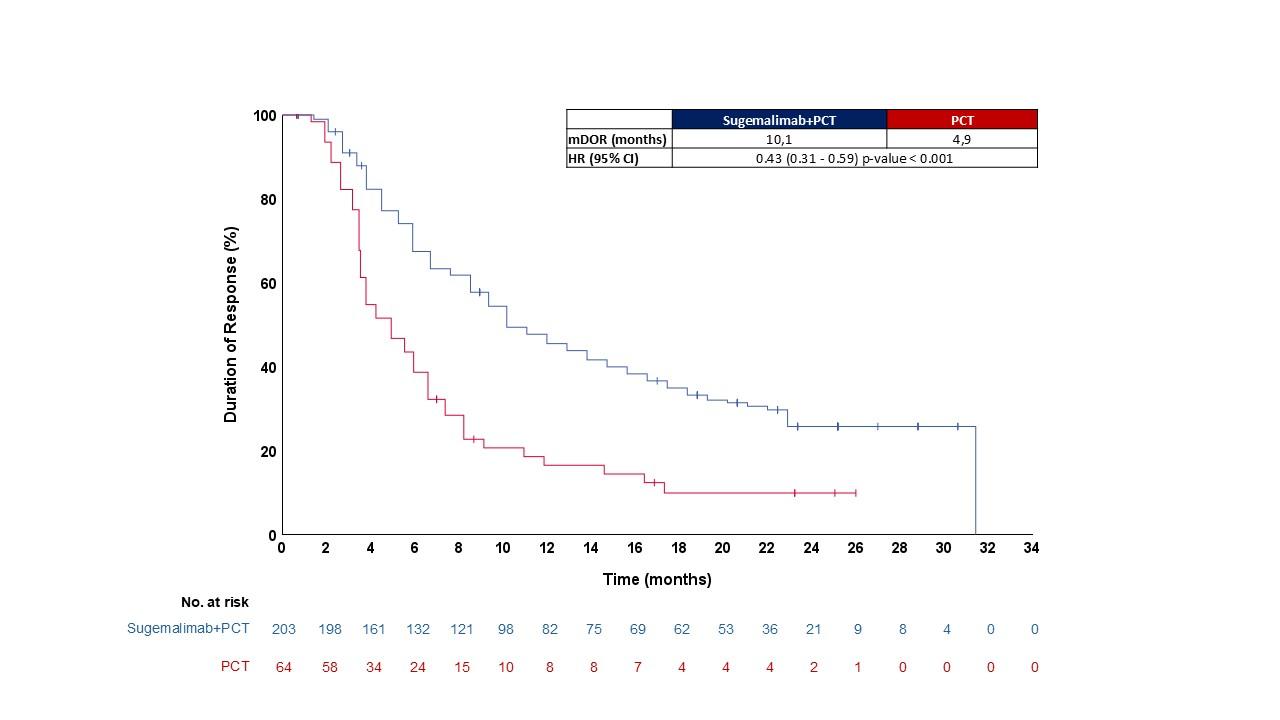 |
| IMpower130 | 10.1016/S1470-2045(19)30167-6 | 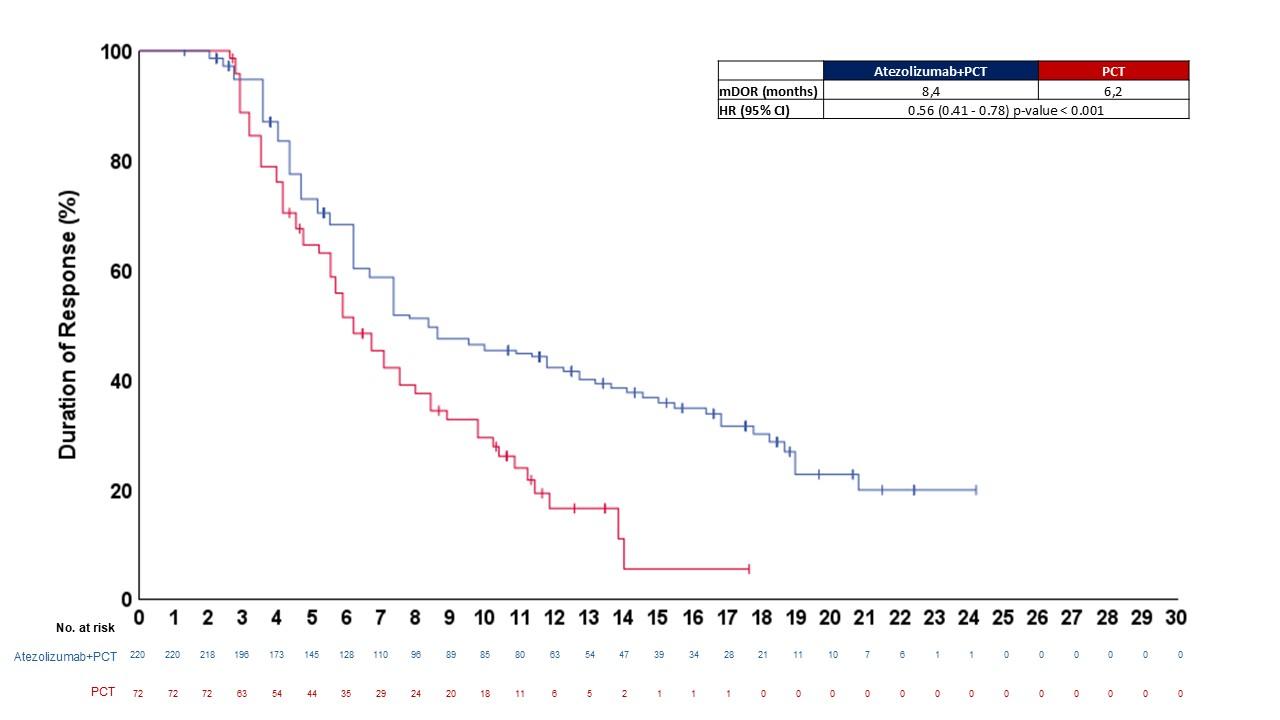 |
| KEYNOTE021 | 10.1016/j.jtho.2020.09.015 | 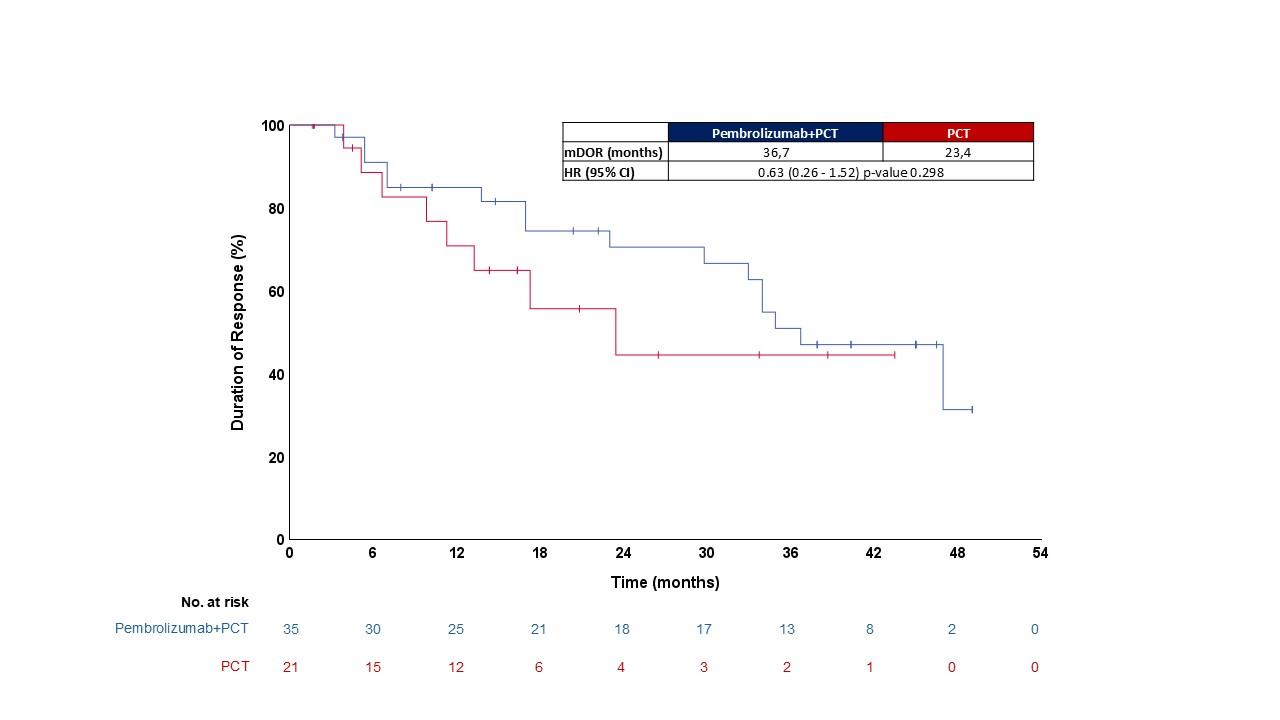 |
| KEYNOTE189 | 10.1200/JCO.22.01989 | 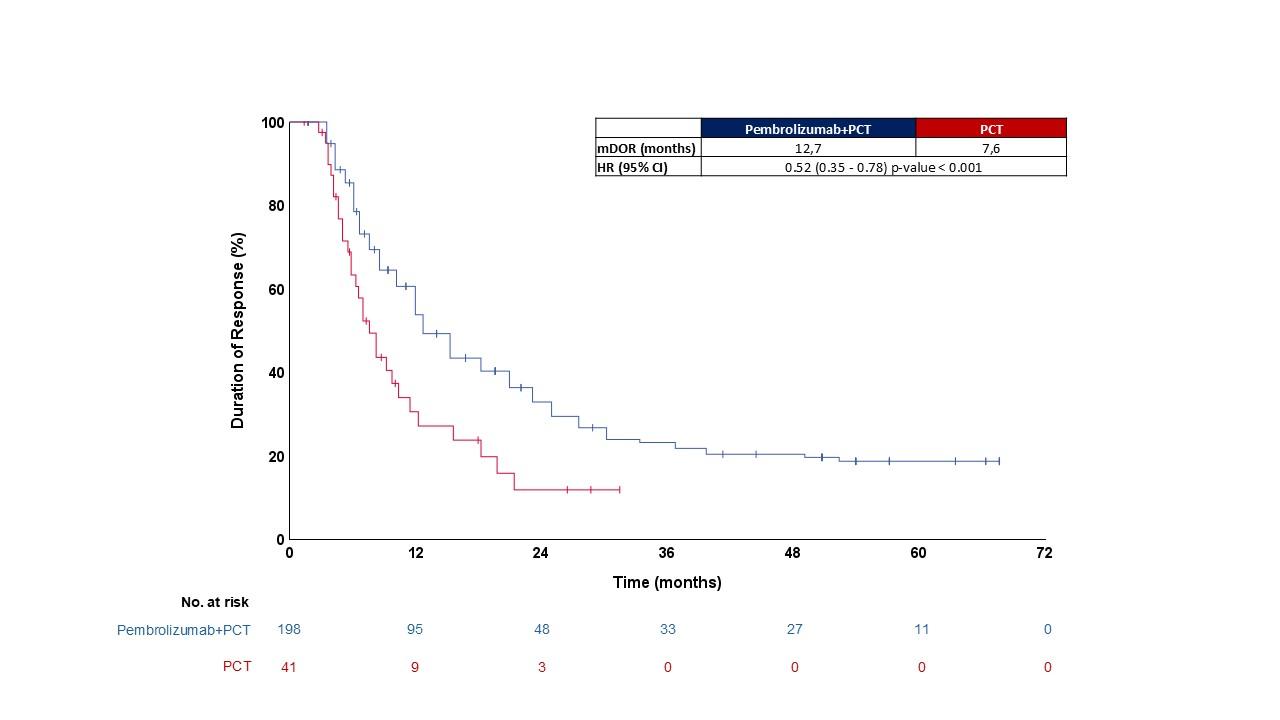 |
| KEYNOTE407 | 10.1200/JCO.22.01990 | 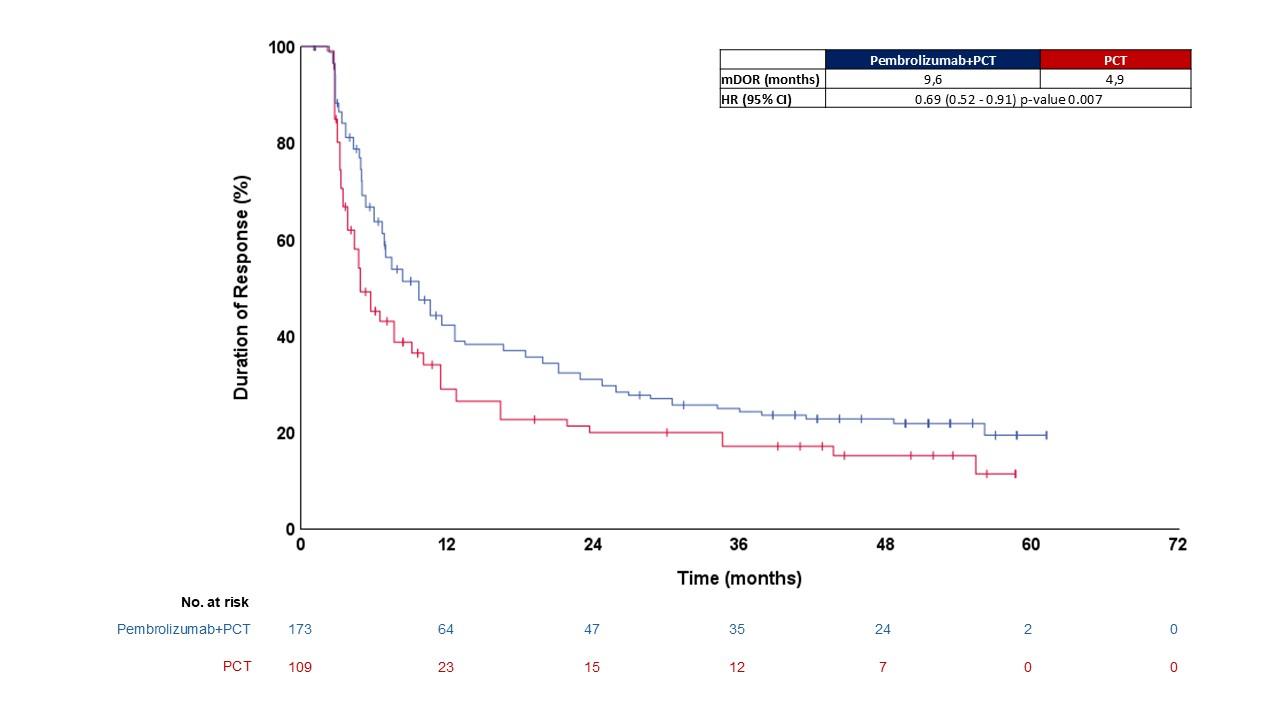 |
| KEYNOTE598 | 10.1200/JCO.20.03579 | 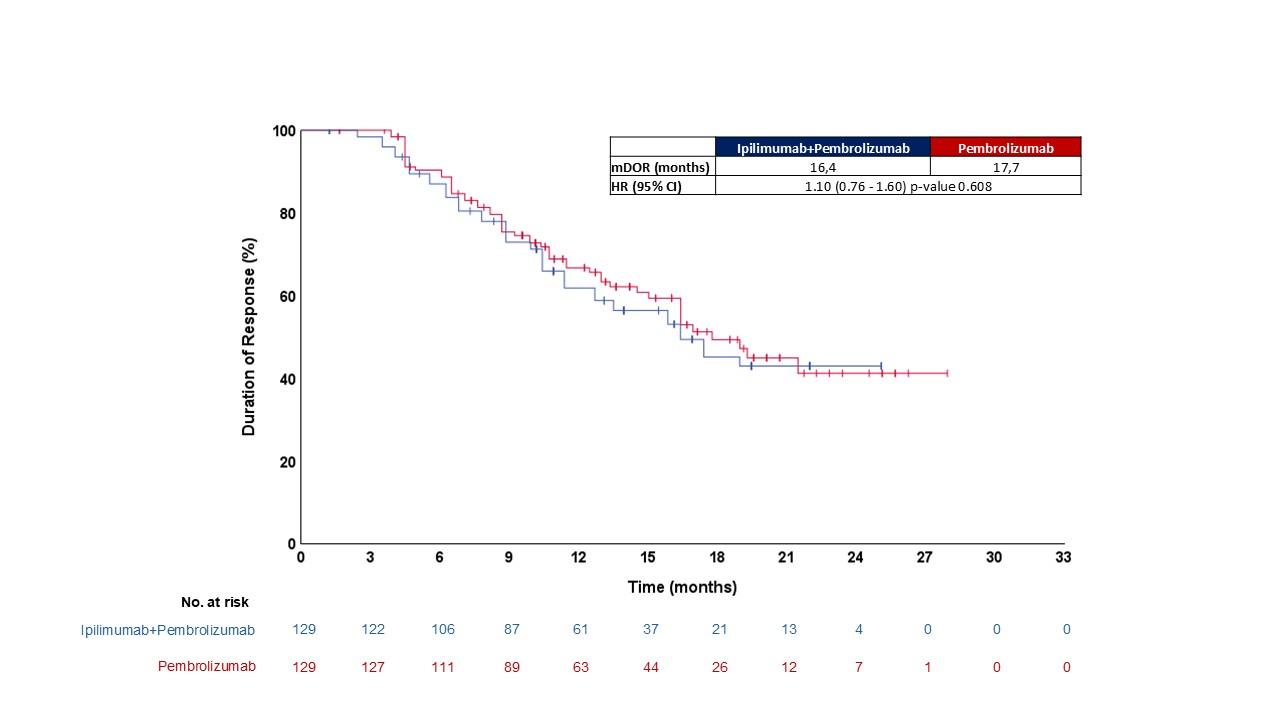 |
| NEPTUNE | 10.1016/j.jtho.2022.09.223 | 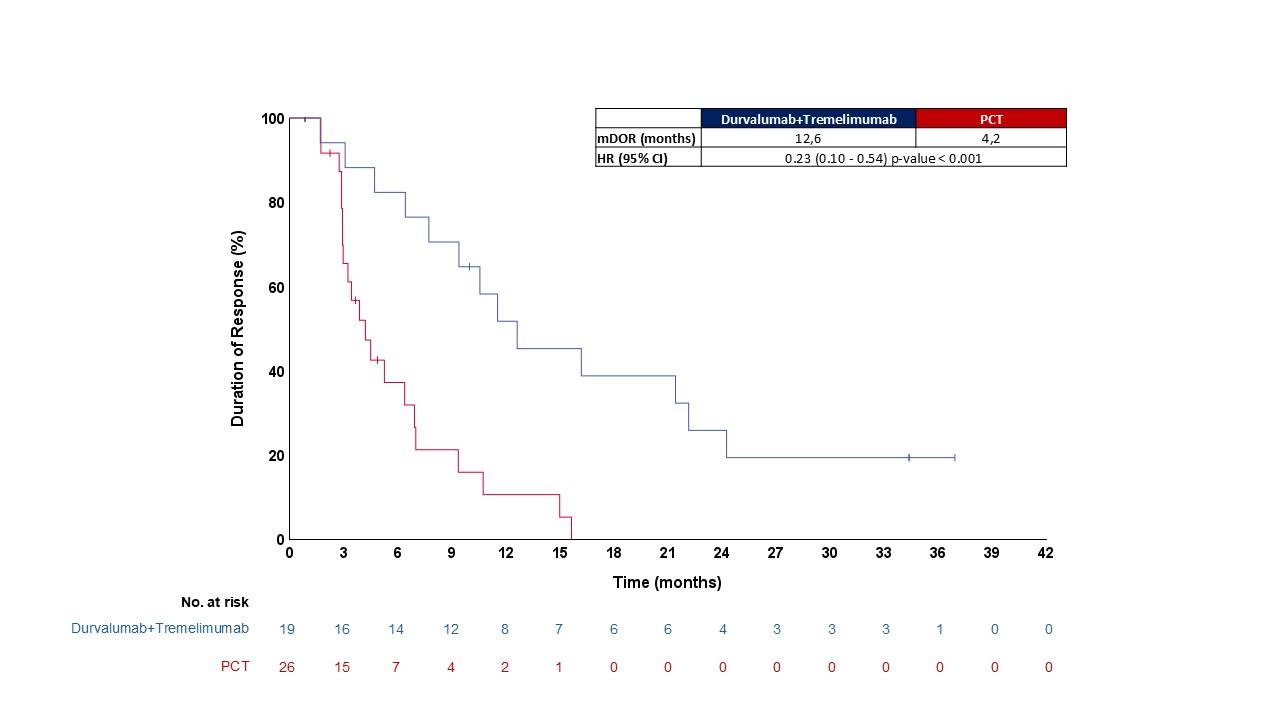 |
| NIPPON | 10.1016/S2213-2600(24)00185-1 | 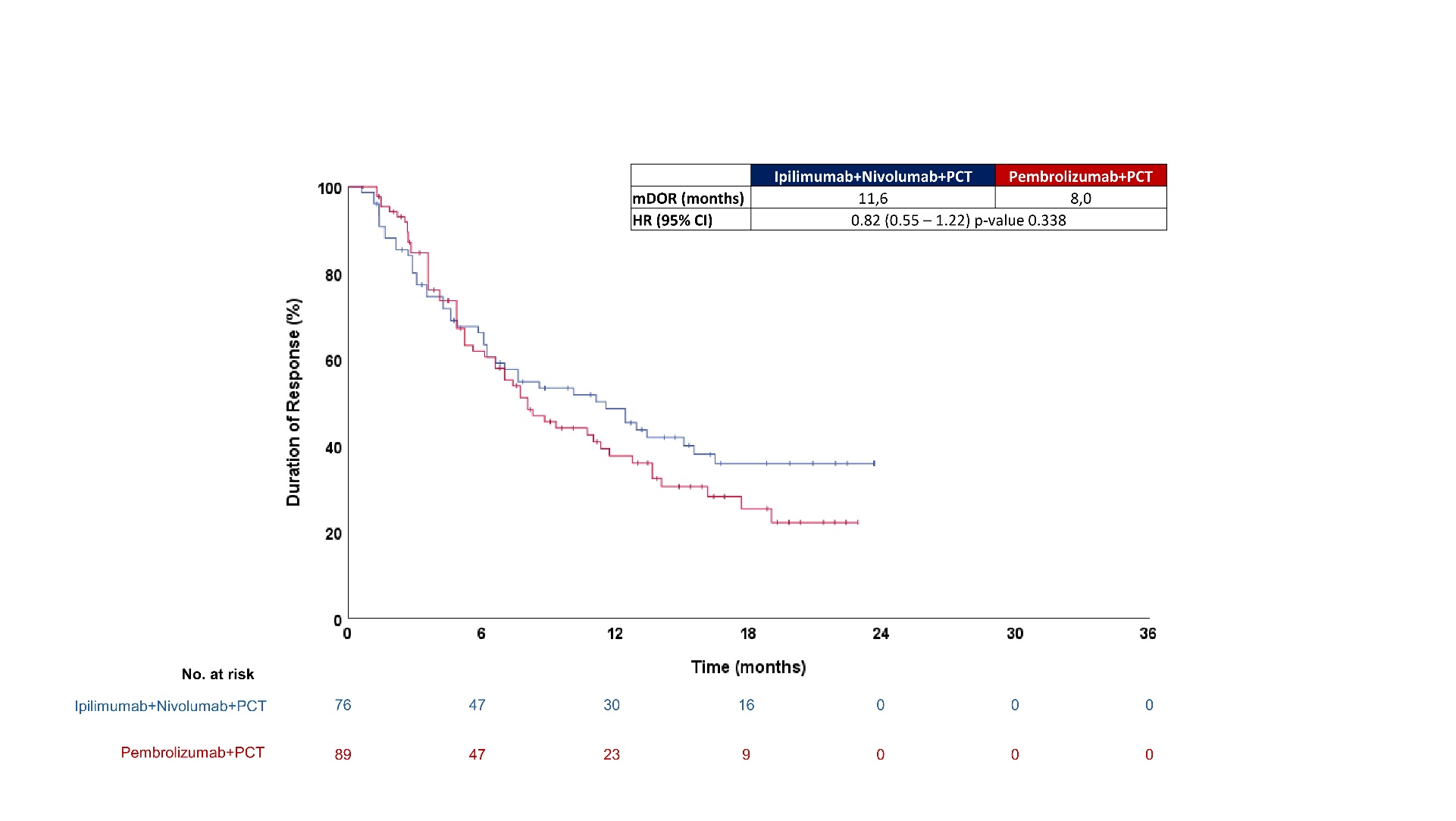 |
| PEARL | 10.1016/j.jtho.2024.10.024 | 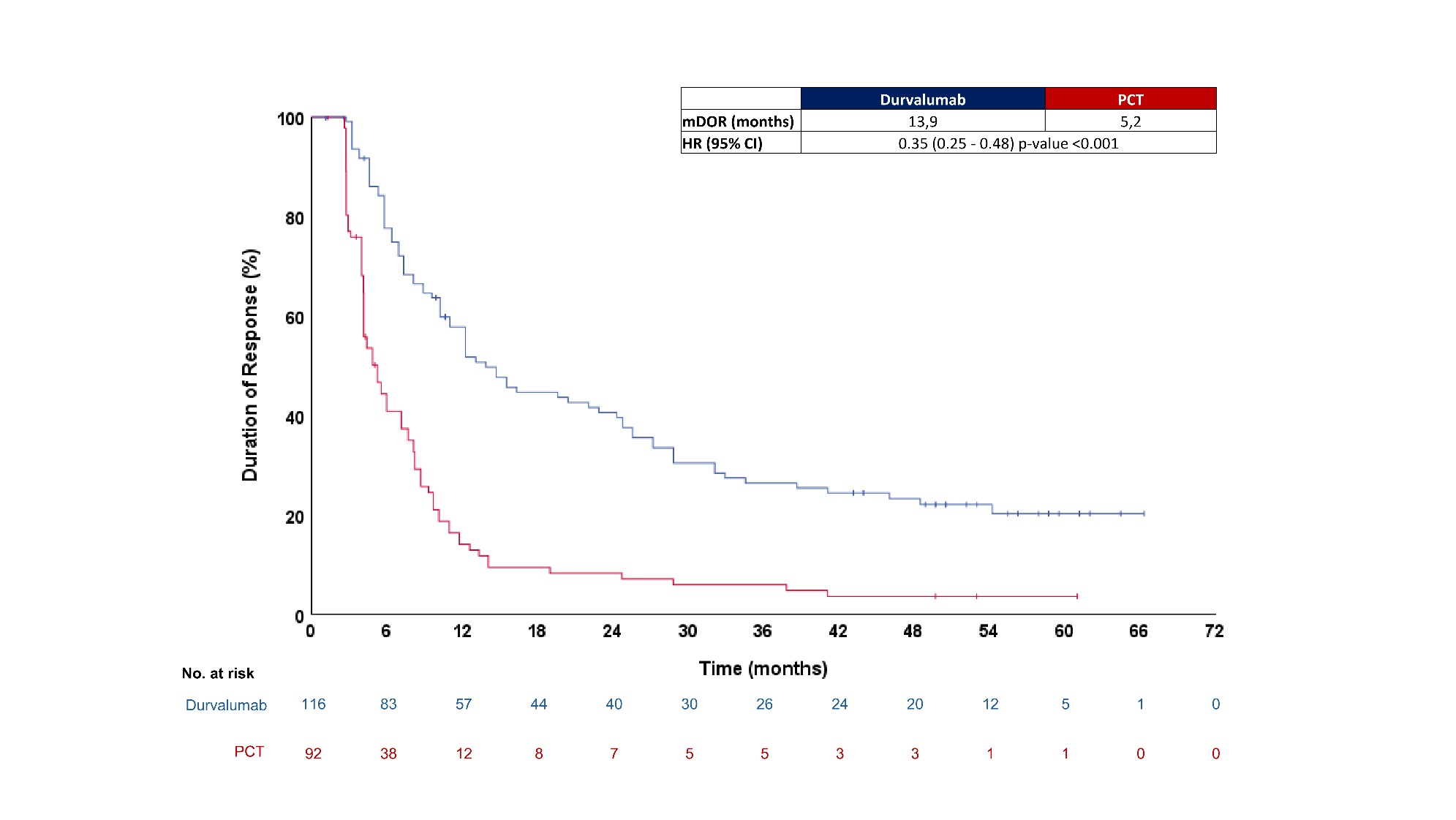 |
| POSEIDON | 10.1200/JCO.22.00975 | 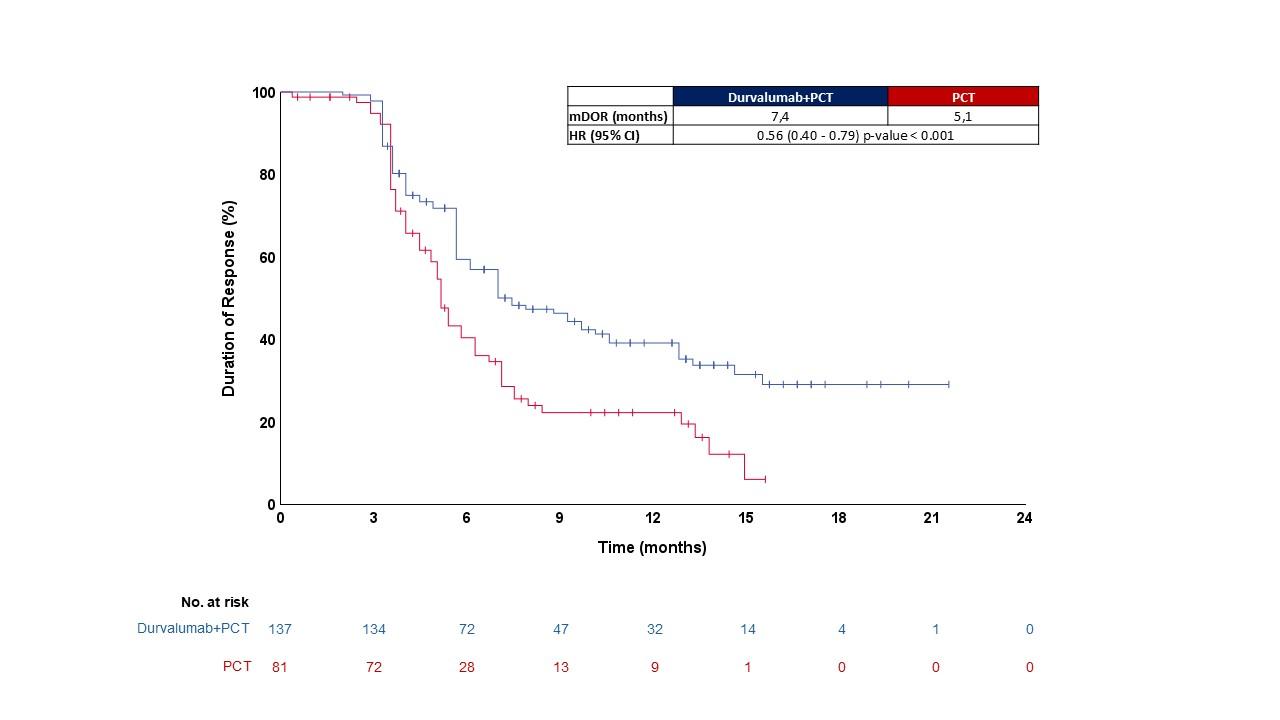  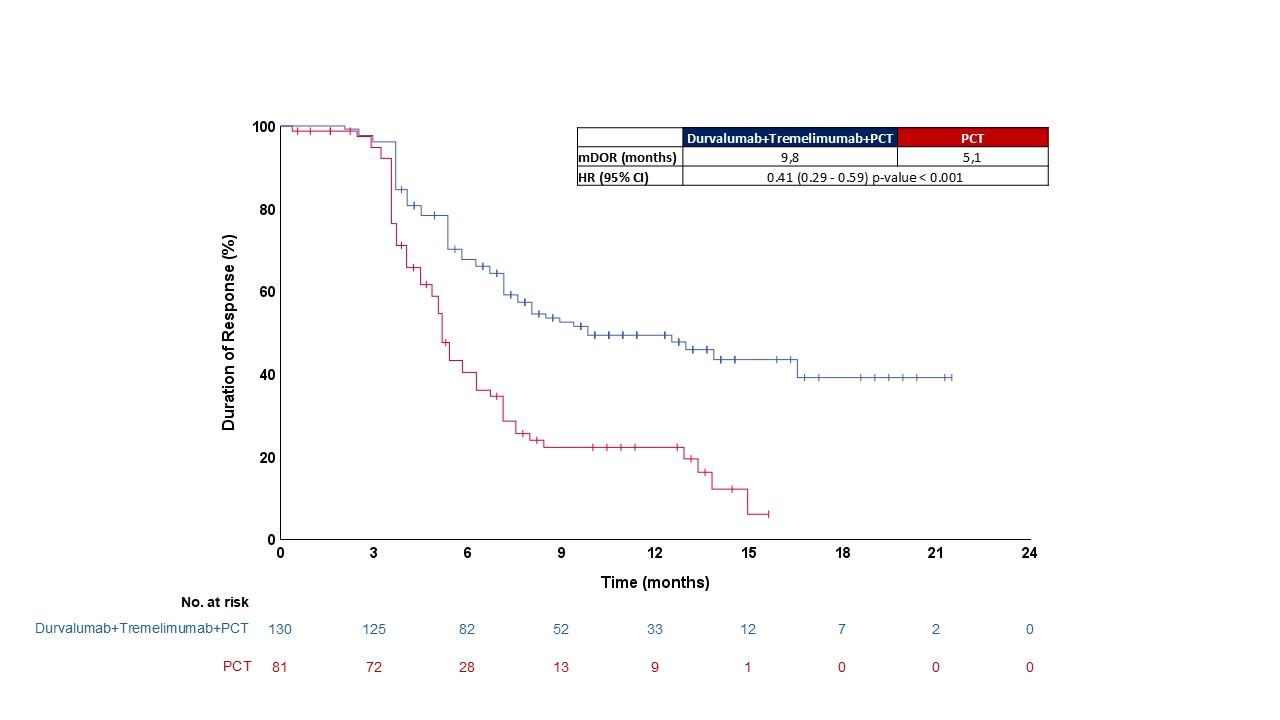 |

**Supplementary Figure 1.** Risk of bias summary percentages across all the included studies.

**
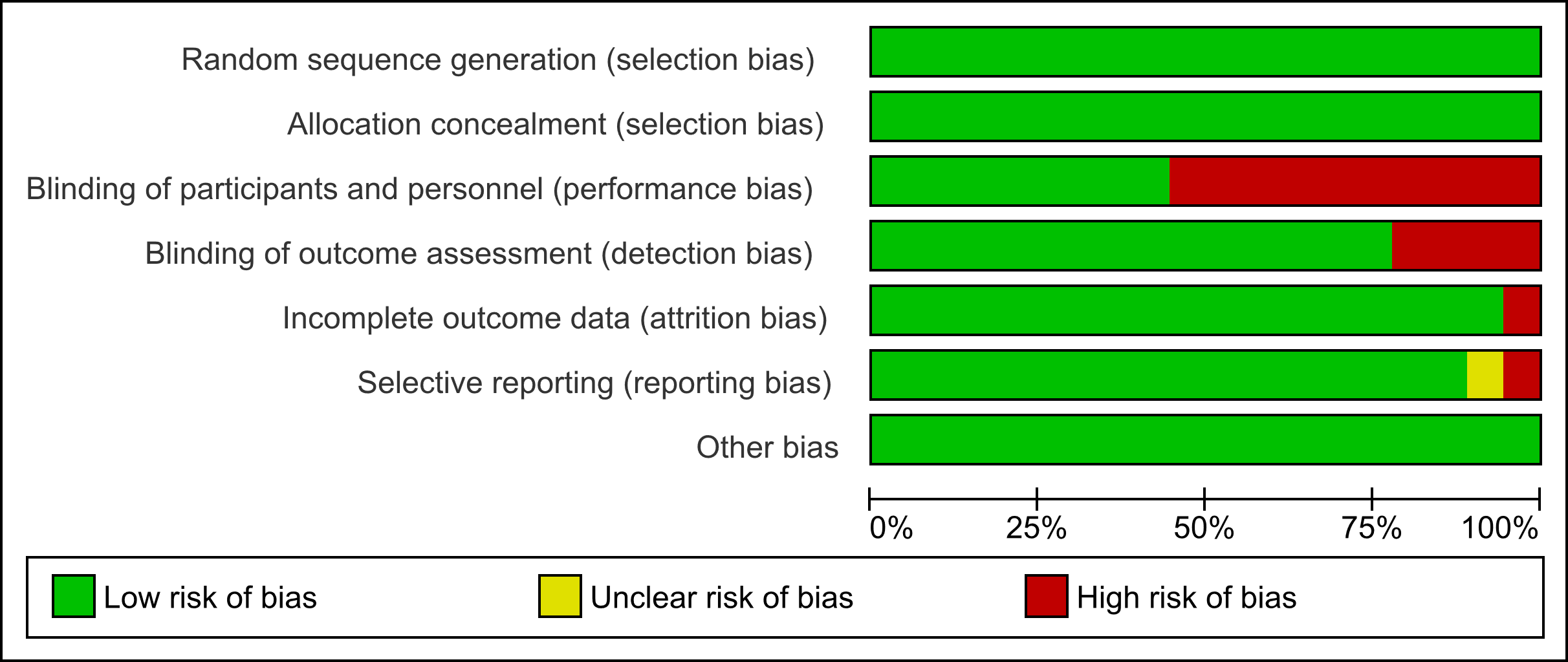
**

**Supplementary Figure 2.** Risk of bias summary for each included study

**
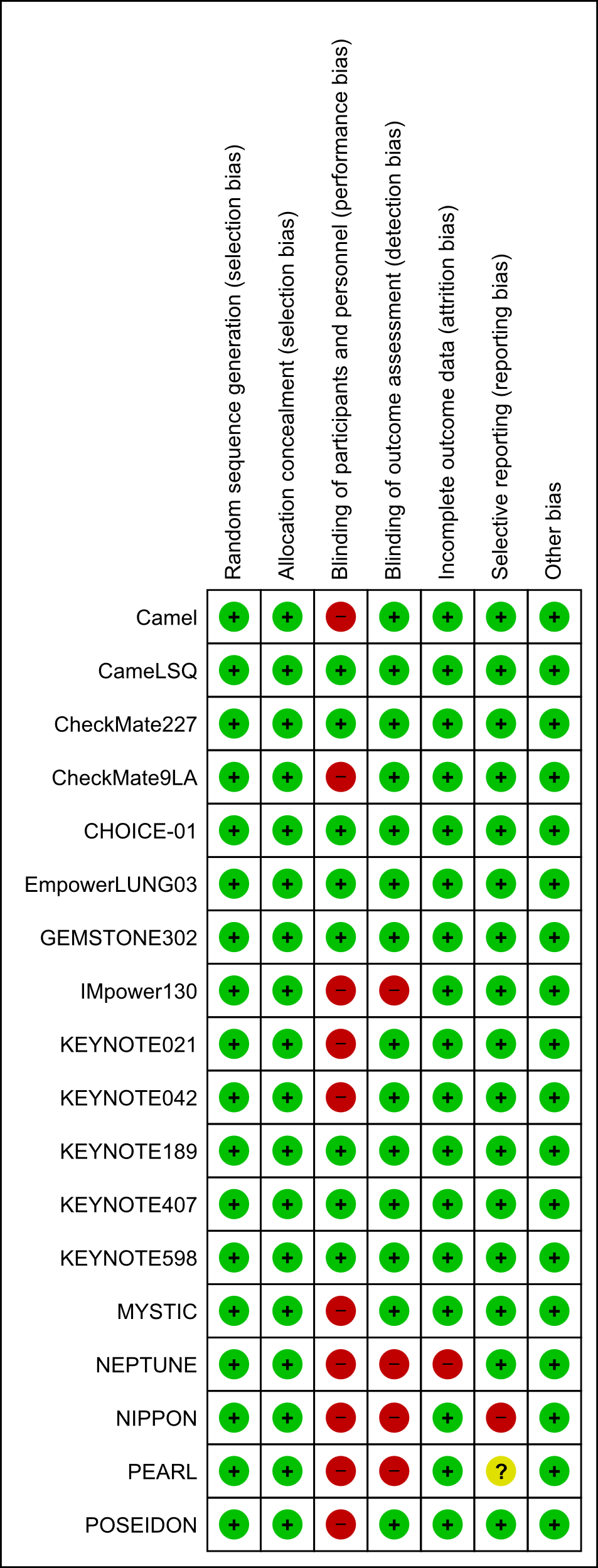
**

**Supplementary Figure 3.** Publication bias assessment by funnel plot for mono-ICI + PCT versus PCT


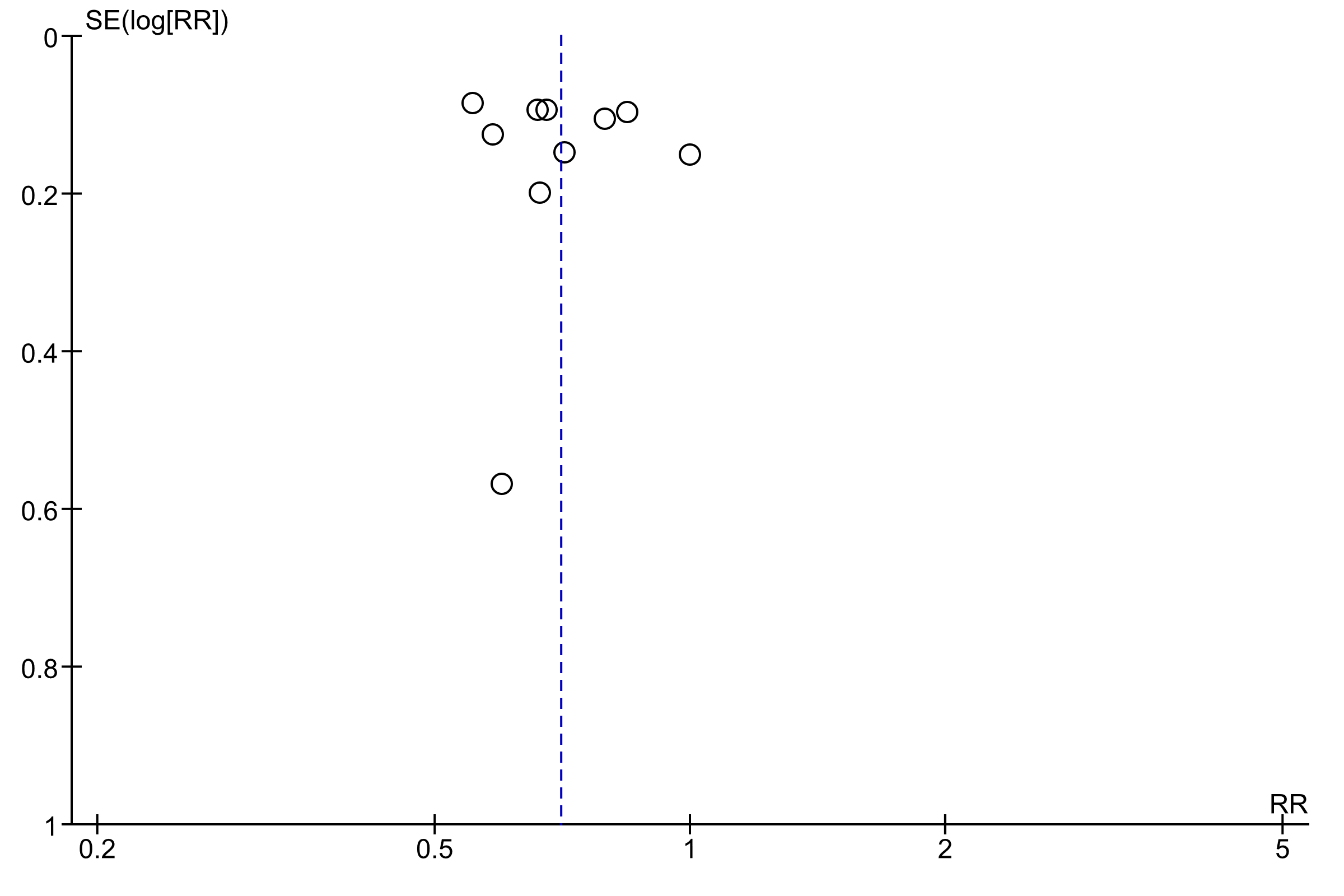


**Supplementary Figure 4.** Acquired resistance rates at 6 (A) and 12 (B) months according to treatment regimens (17 RCTs).

(A)


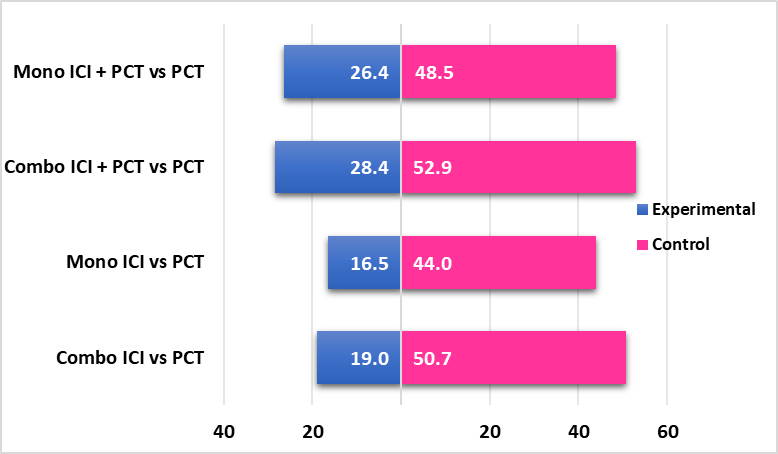


(B)


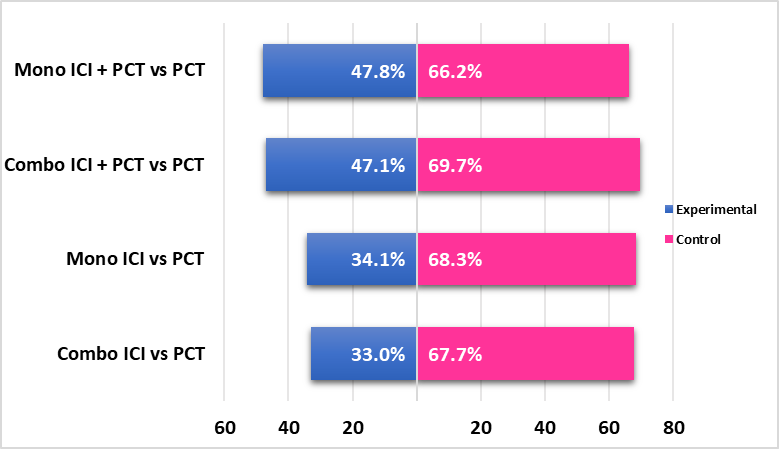


**Supplementary Figure 5.** Comparison of AR risk at 6 months between mono-ICI +PCT versus PCT (A). combo-ICI + PCT versus PCT (B). mono-ICI versus PCT (C). combo-ICI versus PCT (D).

(A)


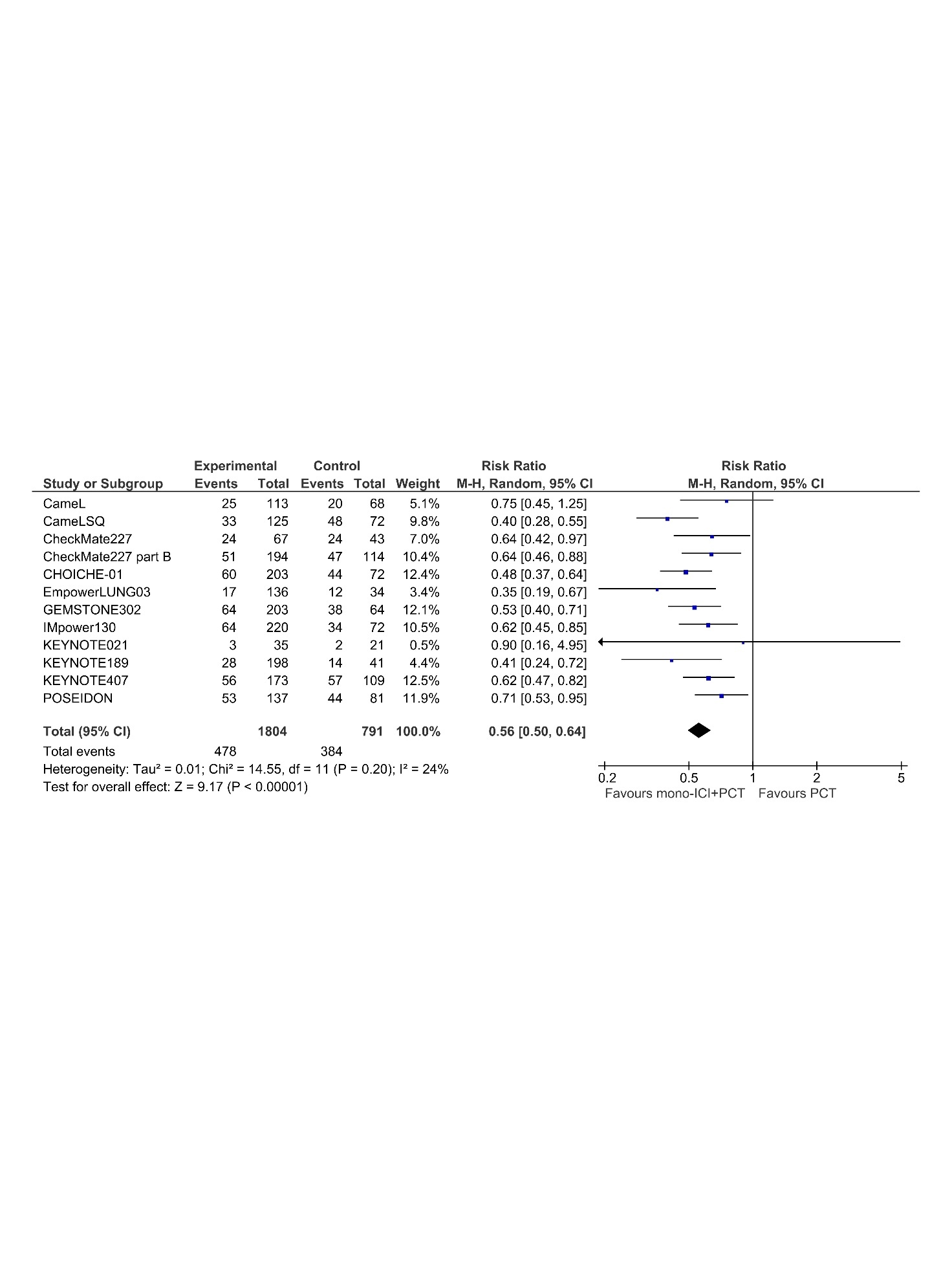

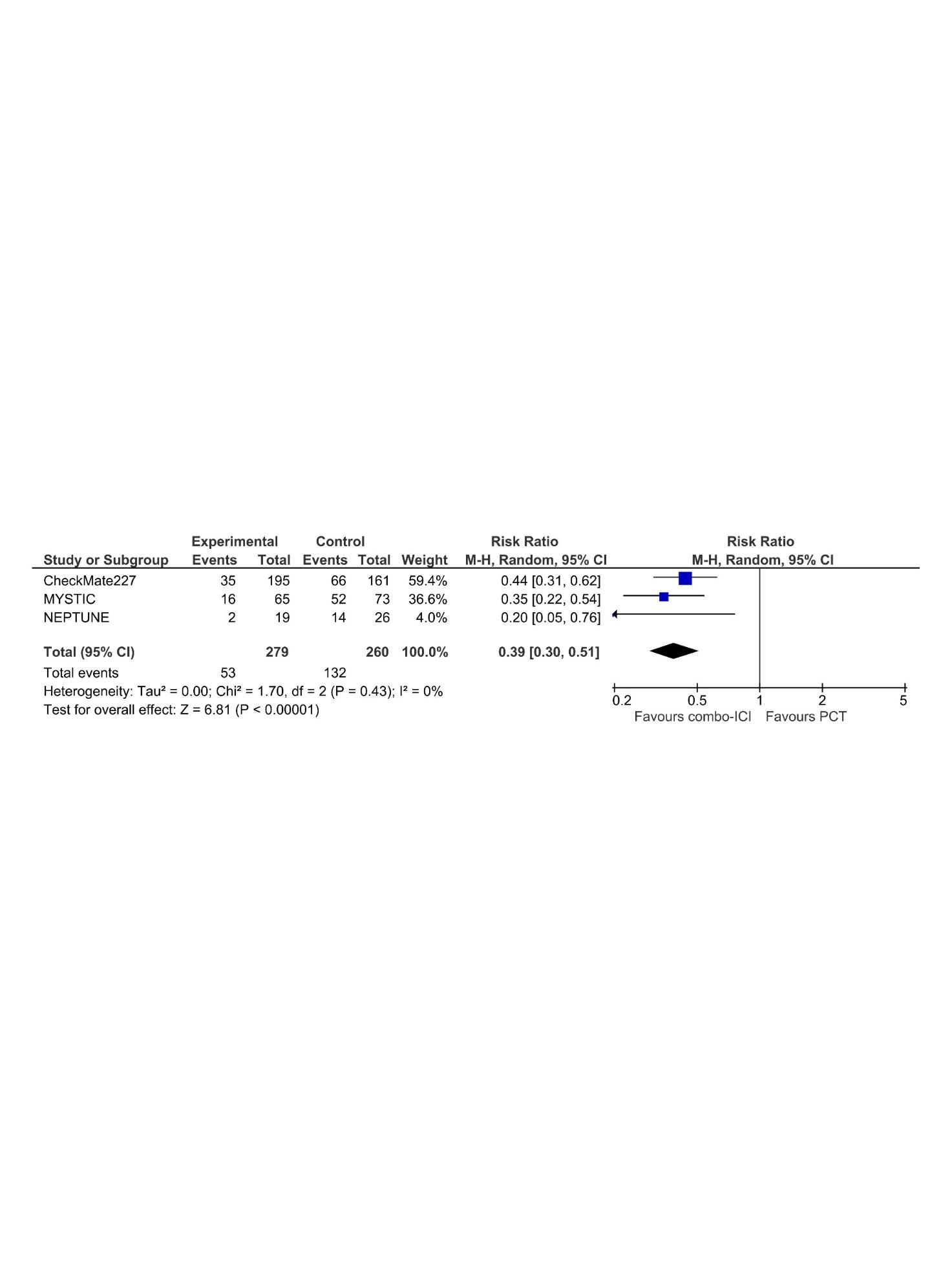


(B)


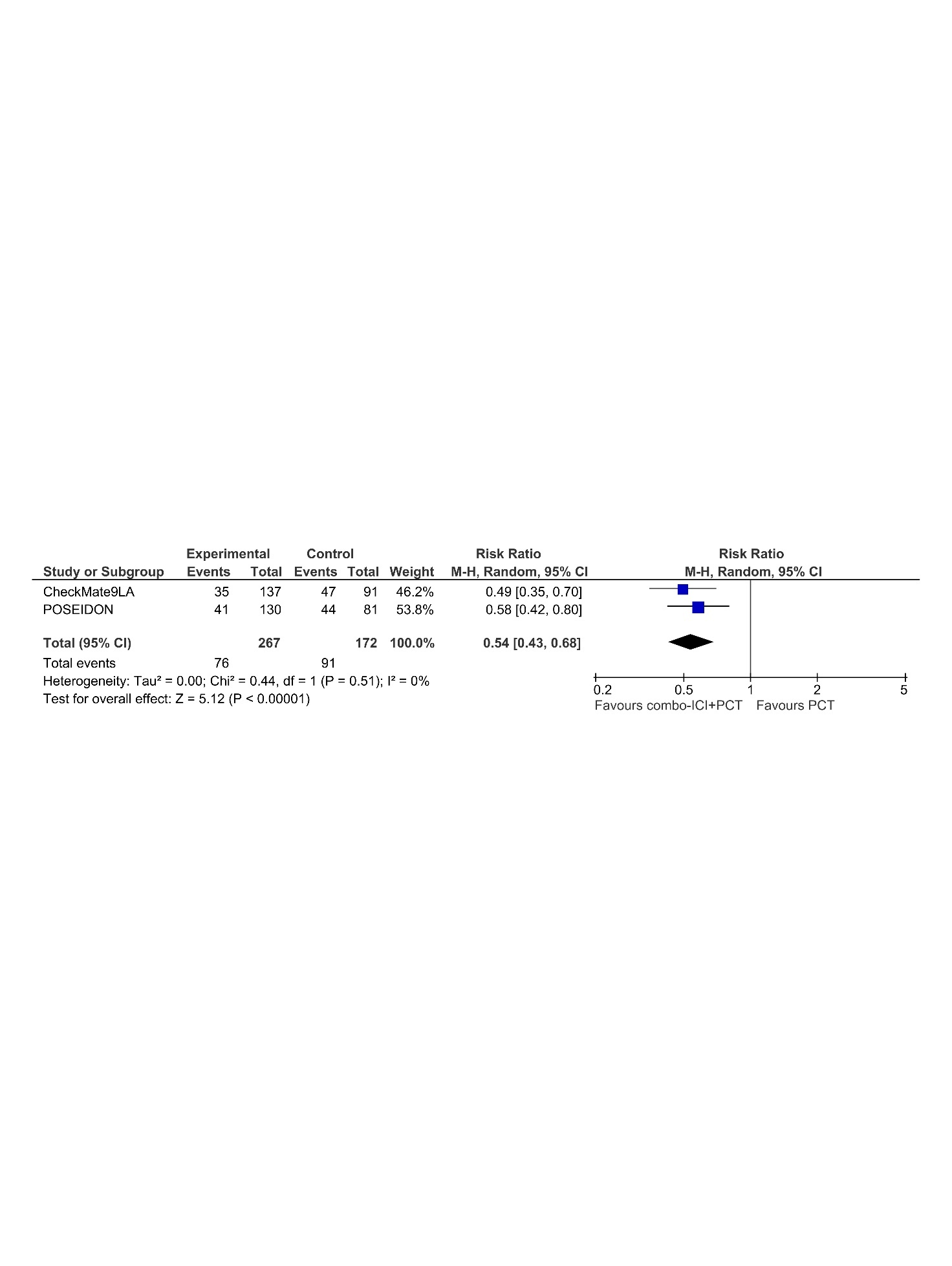


(C)


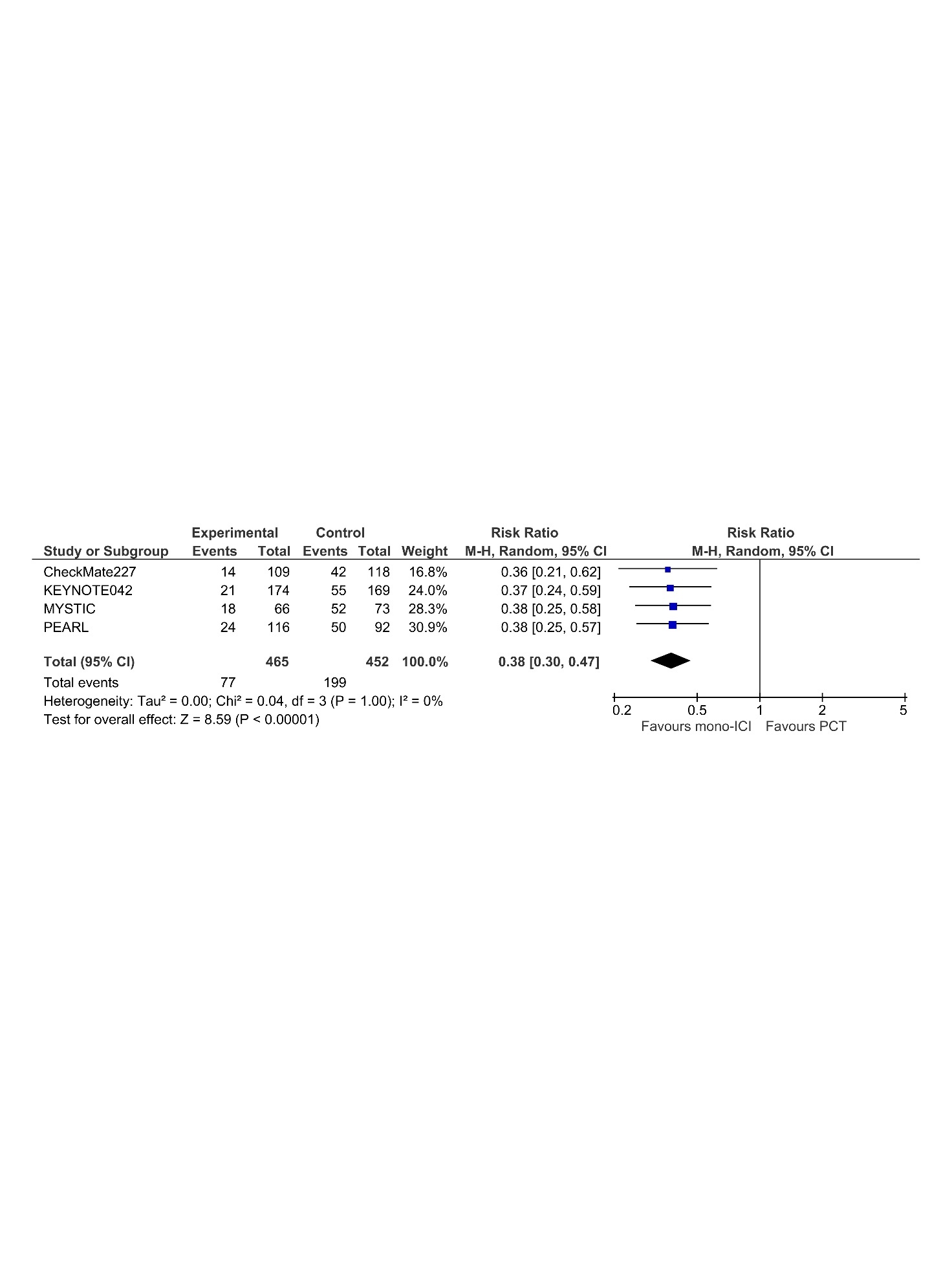


(D)

**Supplementary Figure 6.** Comparison of AR risk at 12 months between mono-ICI + PCT versus PCT (A). combo-ICI + PCT versus PCT (B). mono-ICI versus PCT (C). combo-ICI versus PCT (D).

(A)


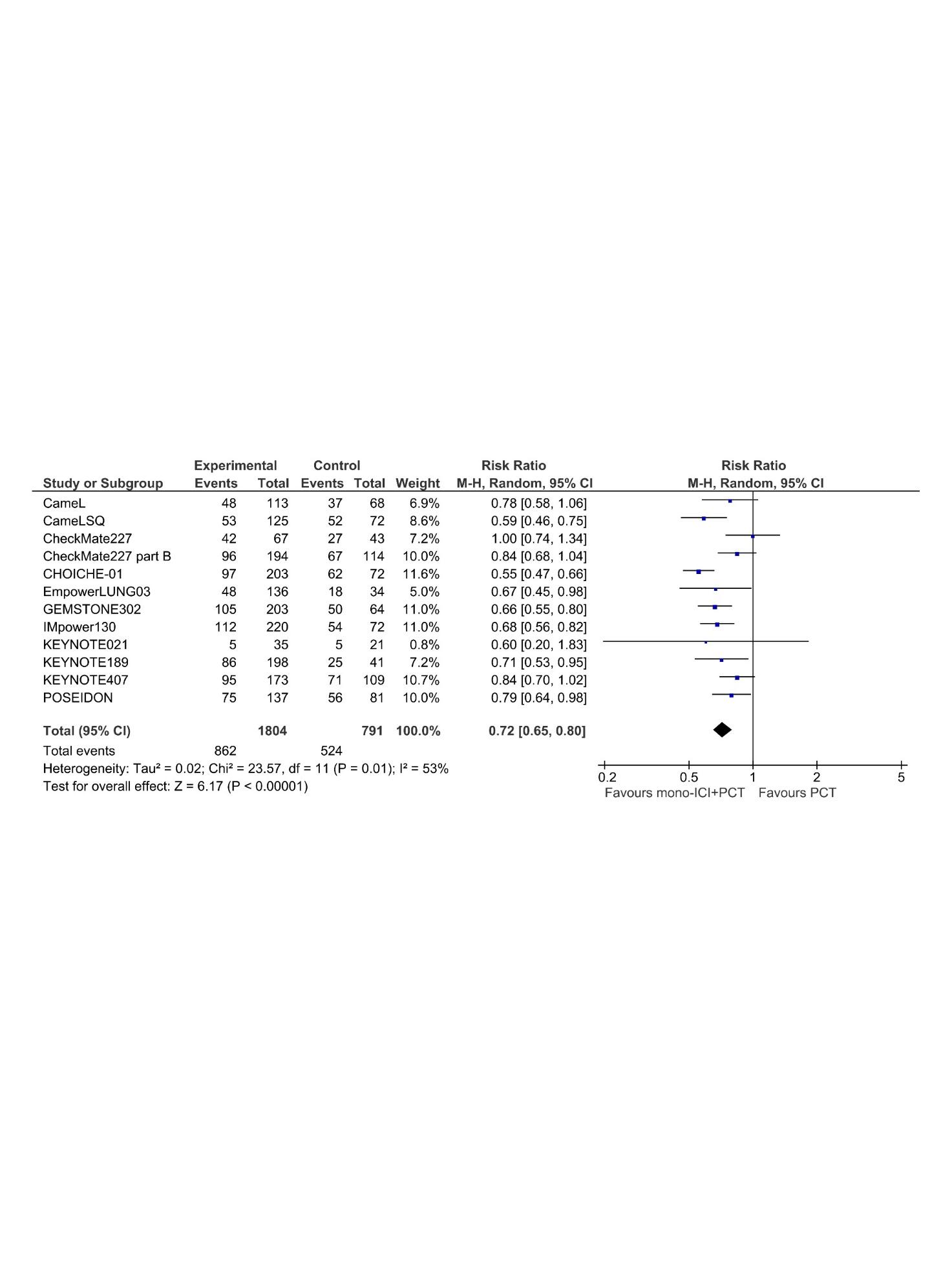


(B)


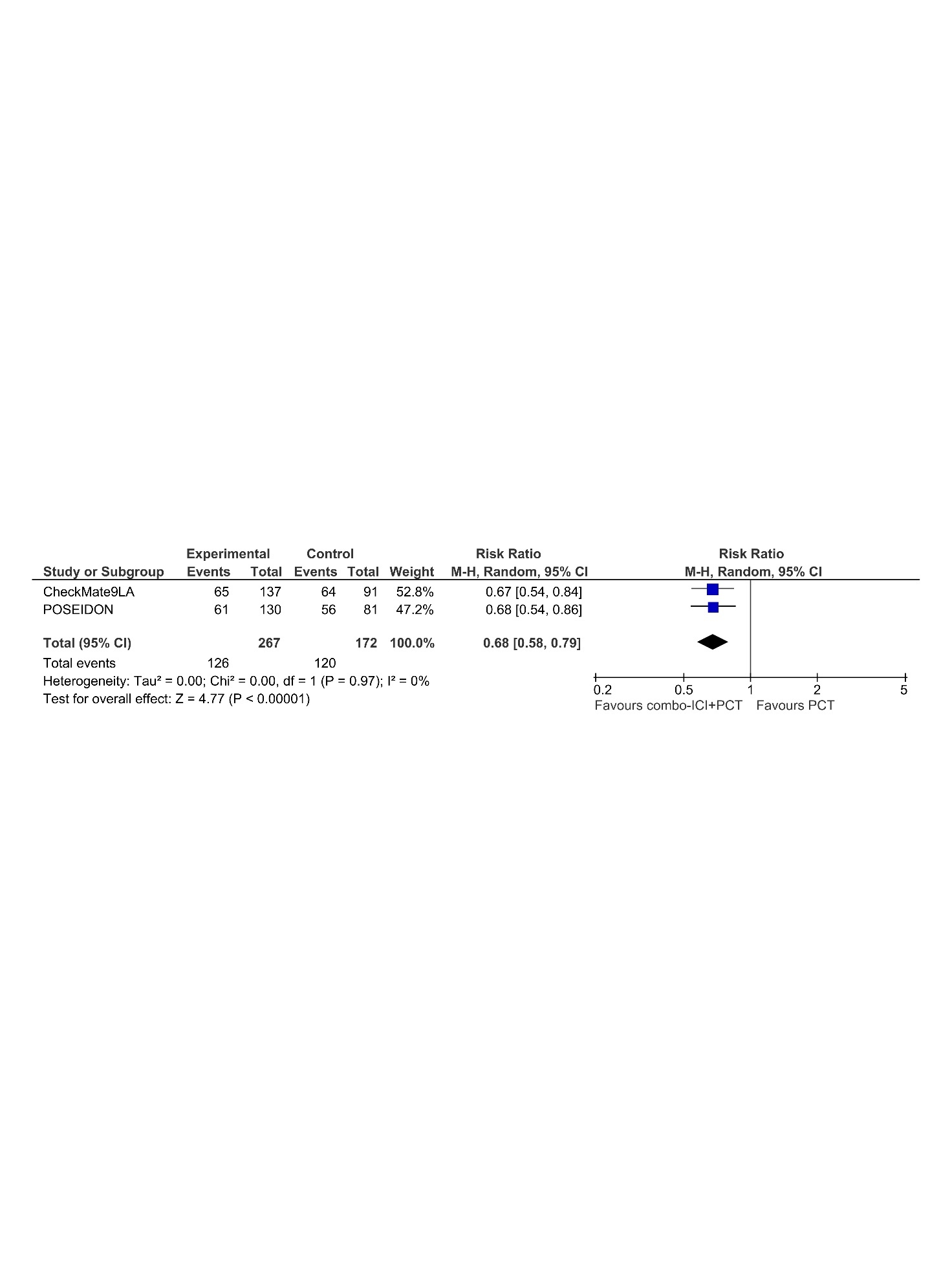


(C)
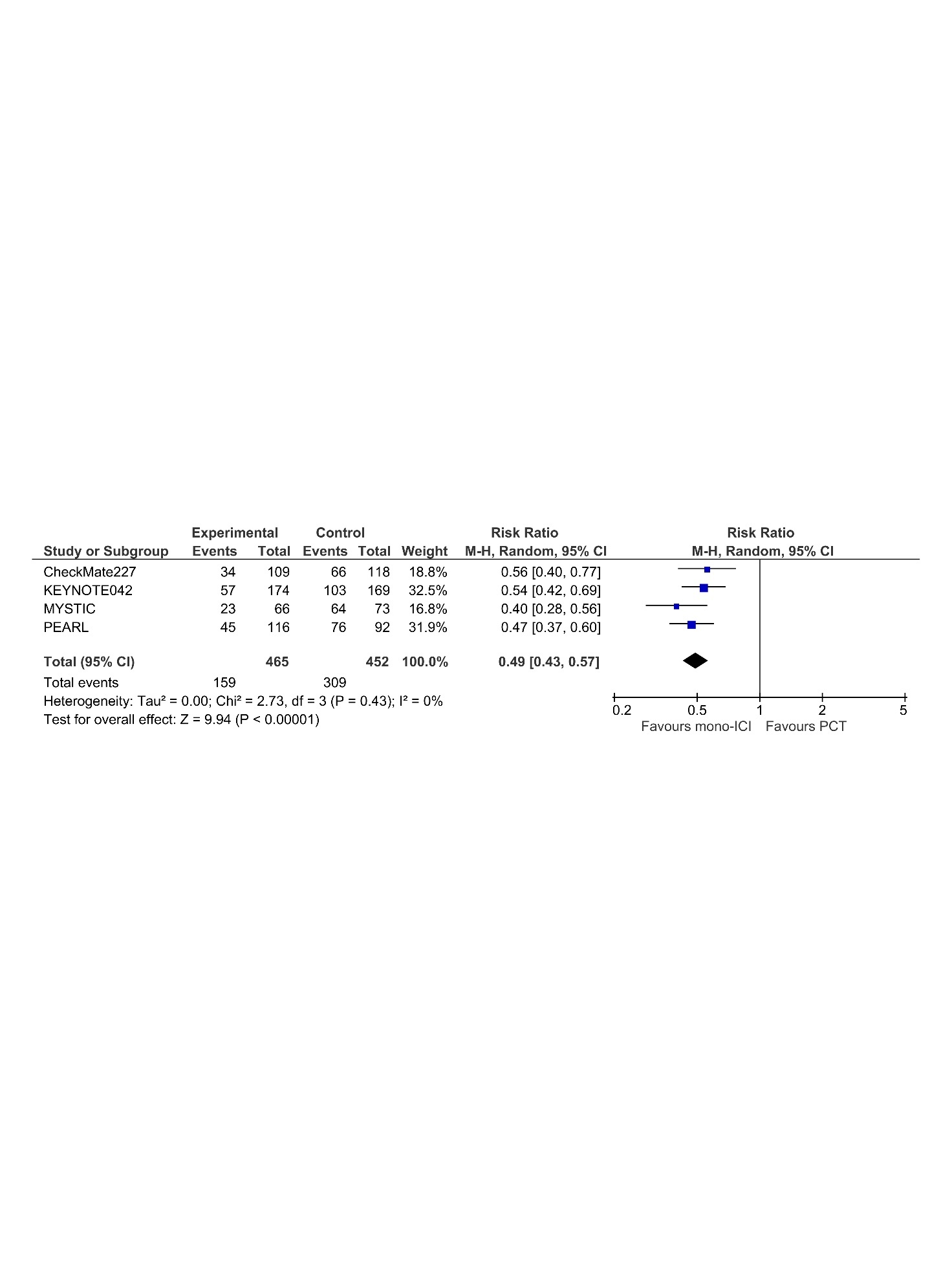


(D)


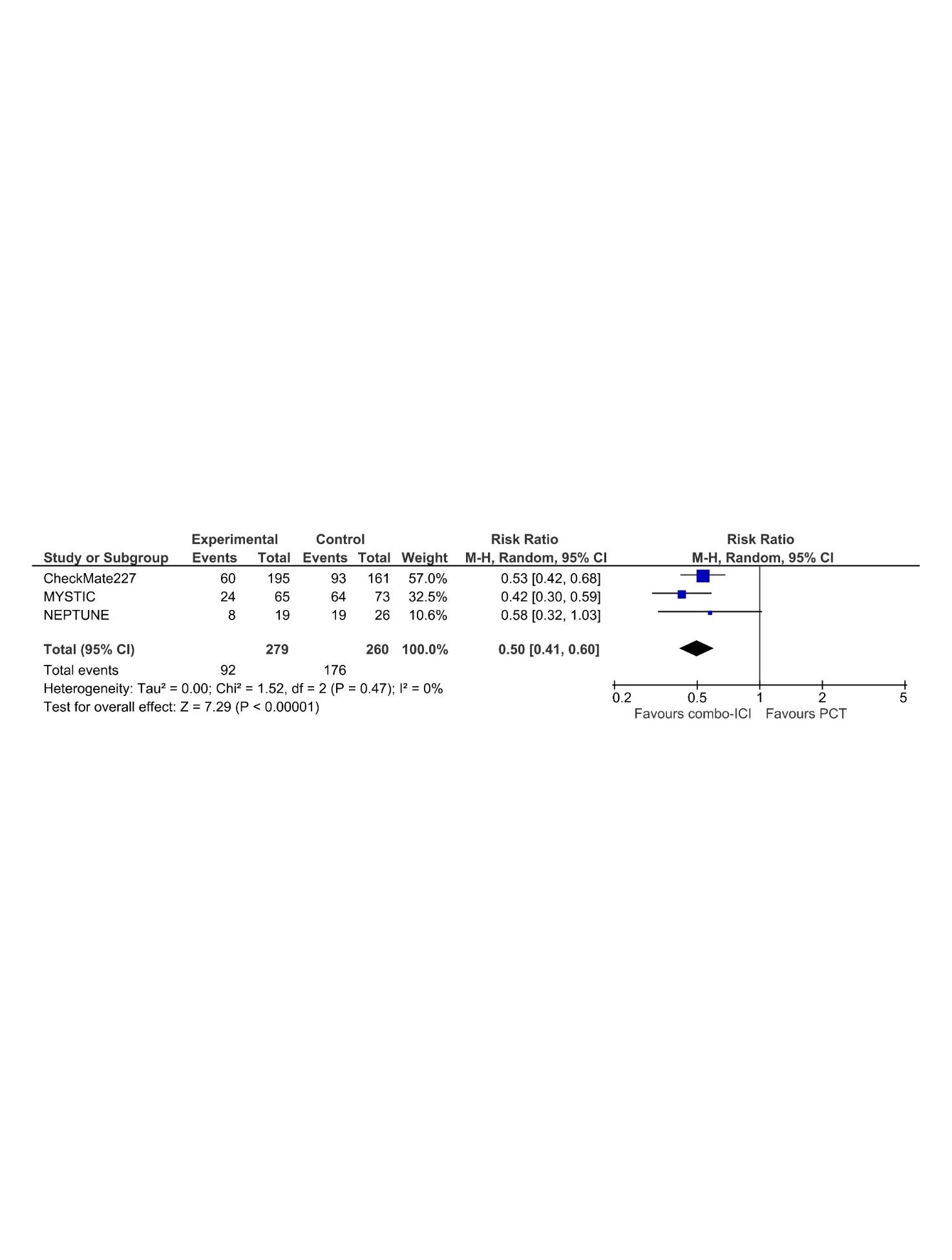


**Supplementary Figure 7.** Censoring imbalance assessment by reverse Kaplan Meier method for the mono-ICI+PCT versus mono-ICI (A). combo-ICI+PCT versus combo-ICI (B). mono-ICI+PCT versus combo-ICI+PCT (C). mono-ICI versus combo-ICI (D).

(A)


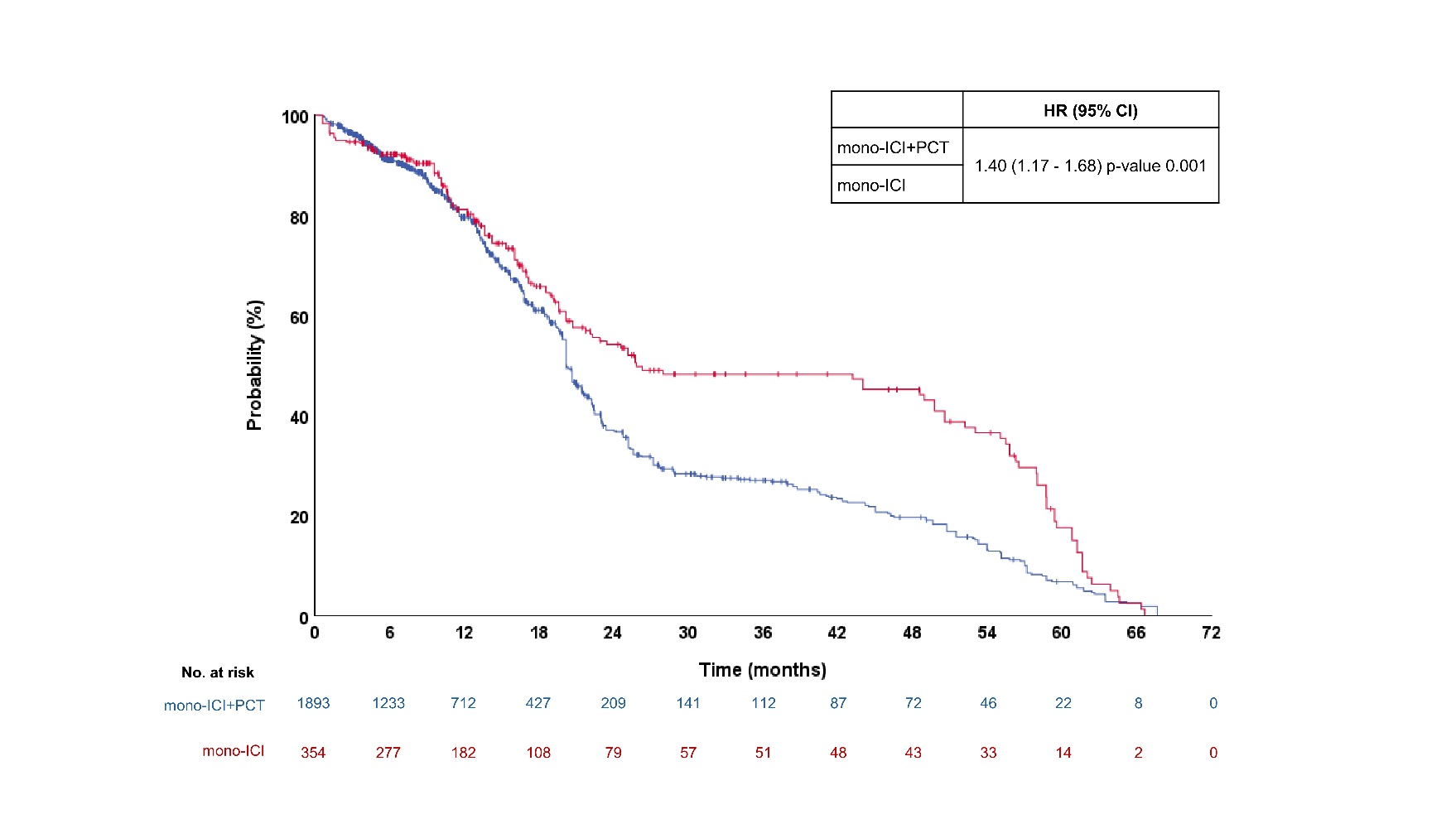


(B)


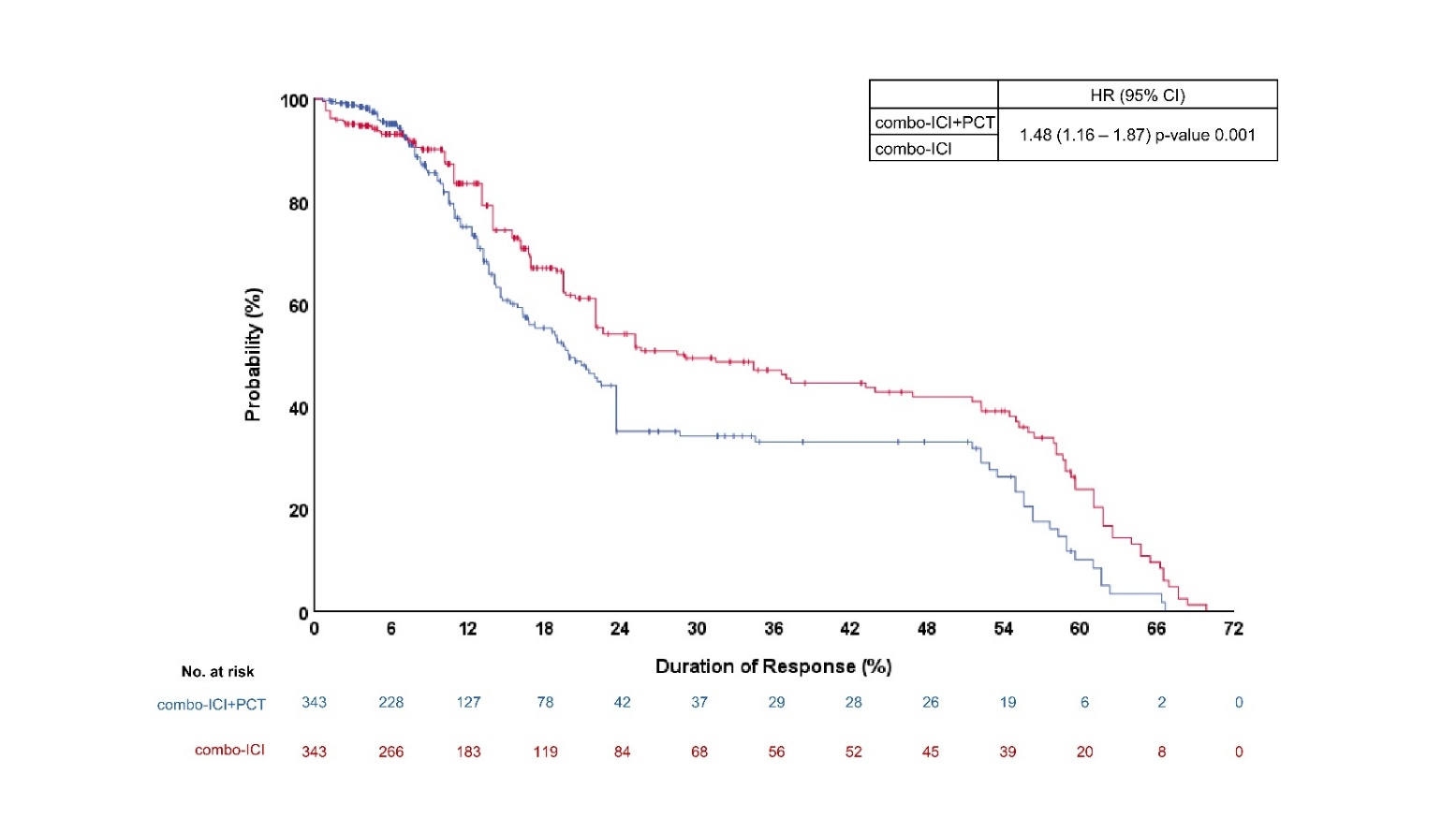


(C)


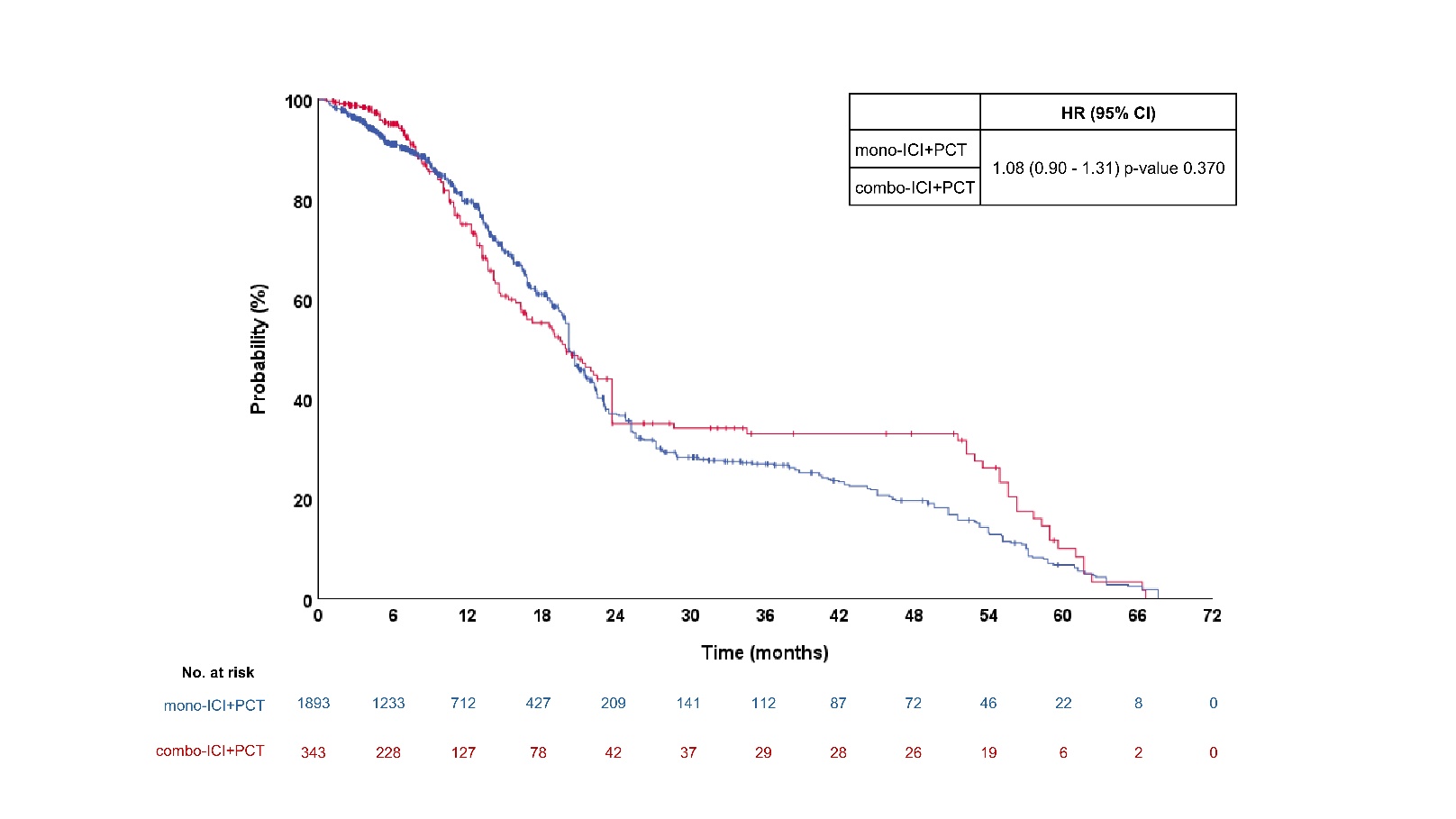


(D)


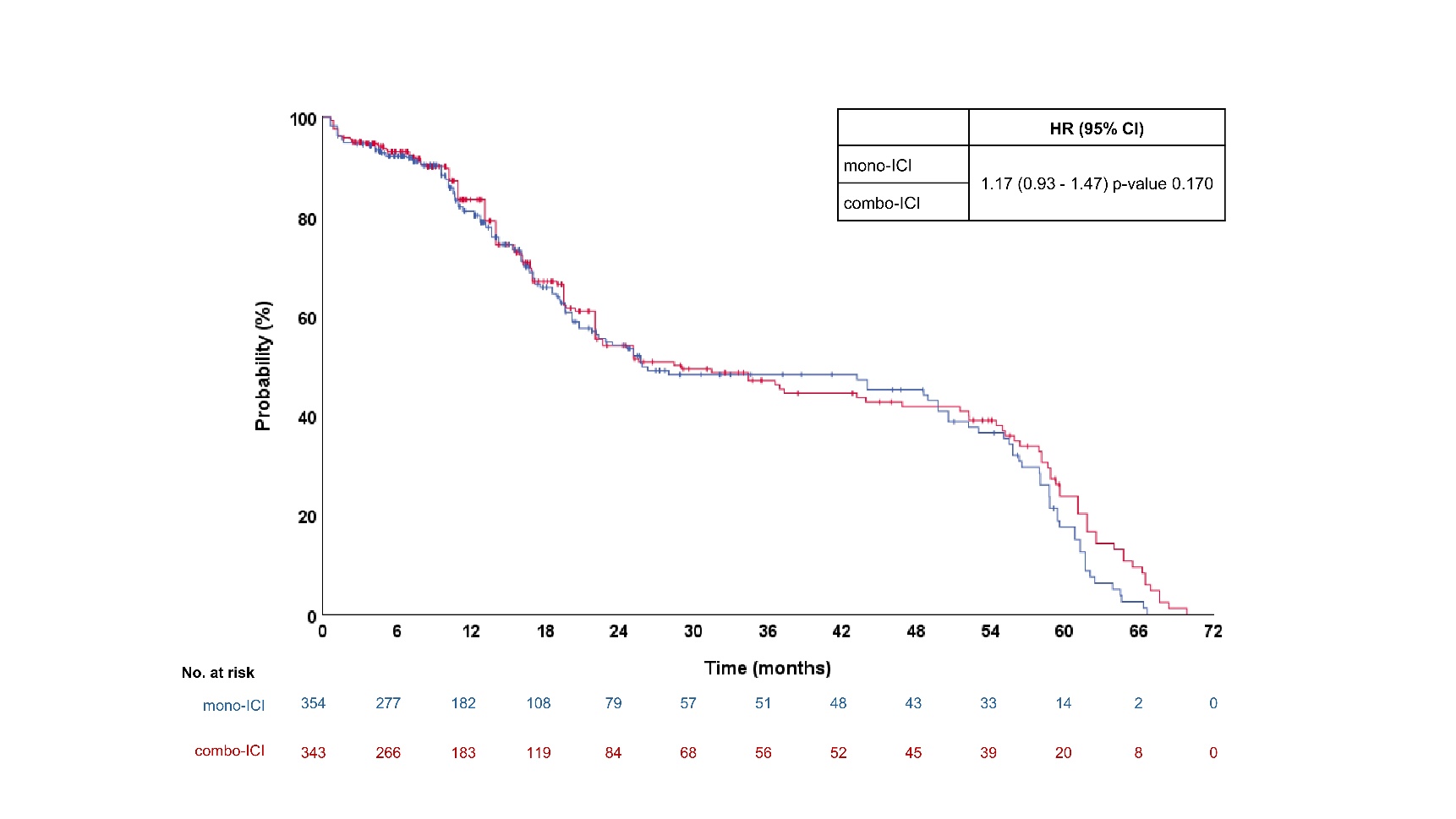


**Supplementary Figure 8.** Sensitivity analysis of mono-ICI+PCT versus mono-ICI (A). combo-ICI+PCT versus combo-ICI (B) eliminating RCTs conducted in PD-L1 and TMB selected populations.

(A)


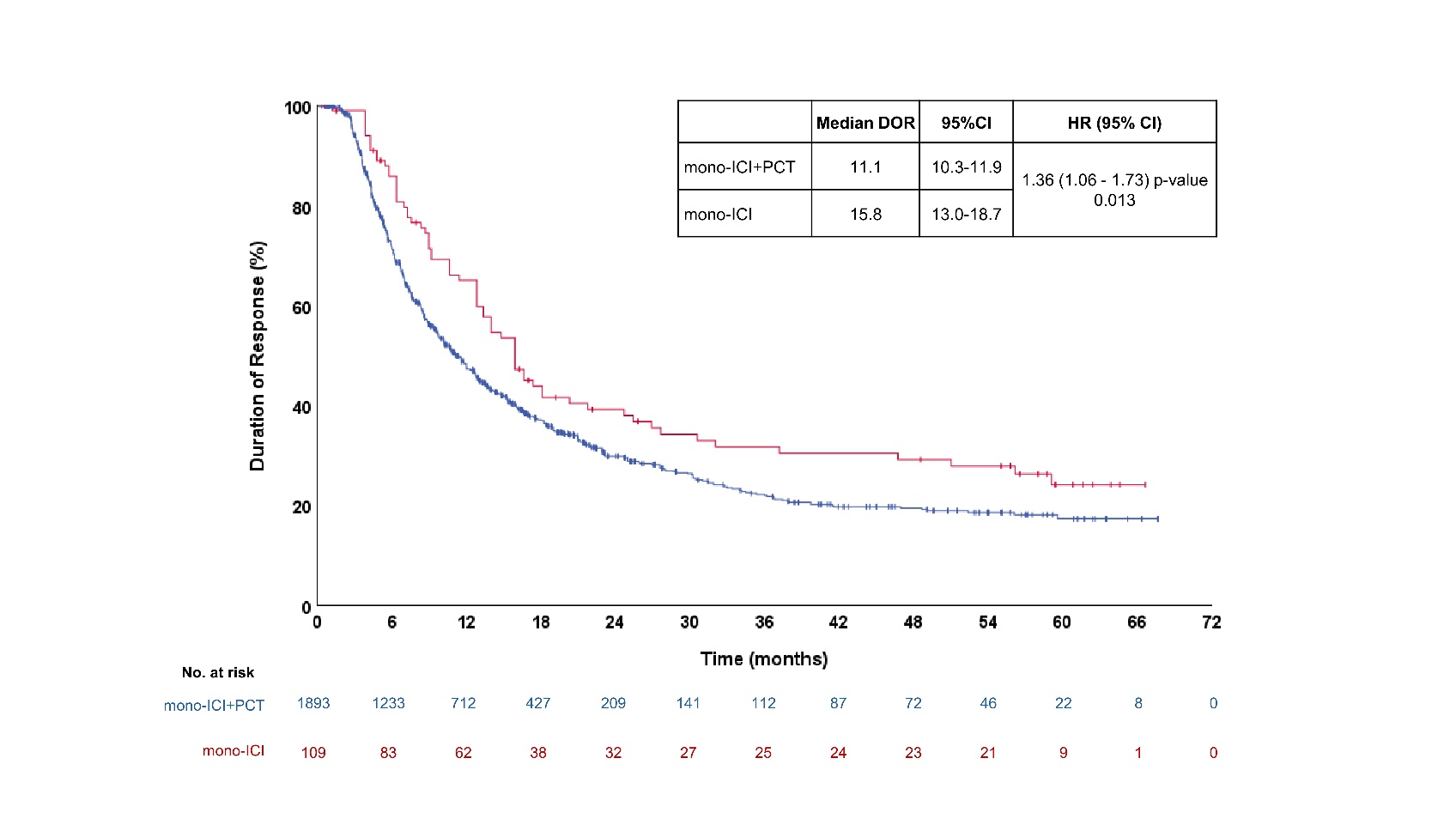


(B)


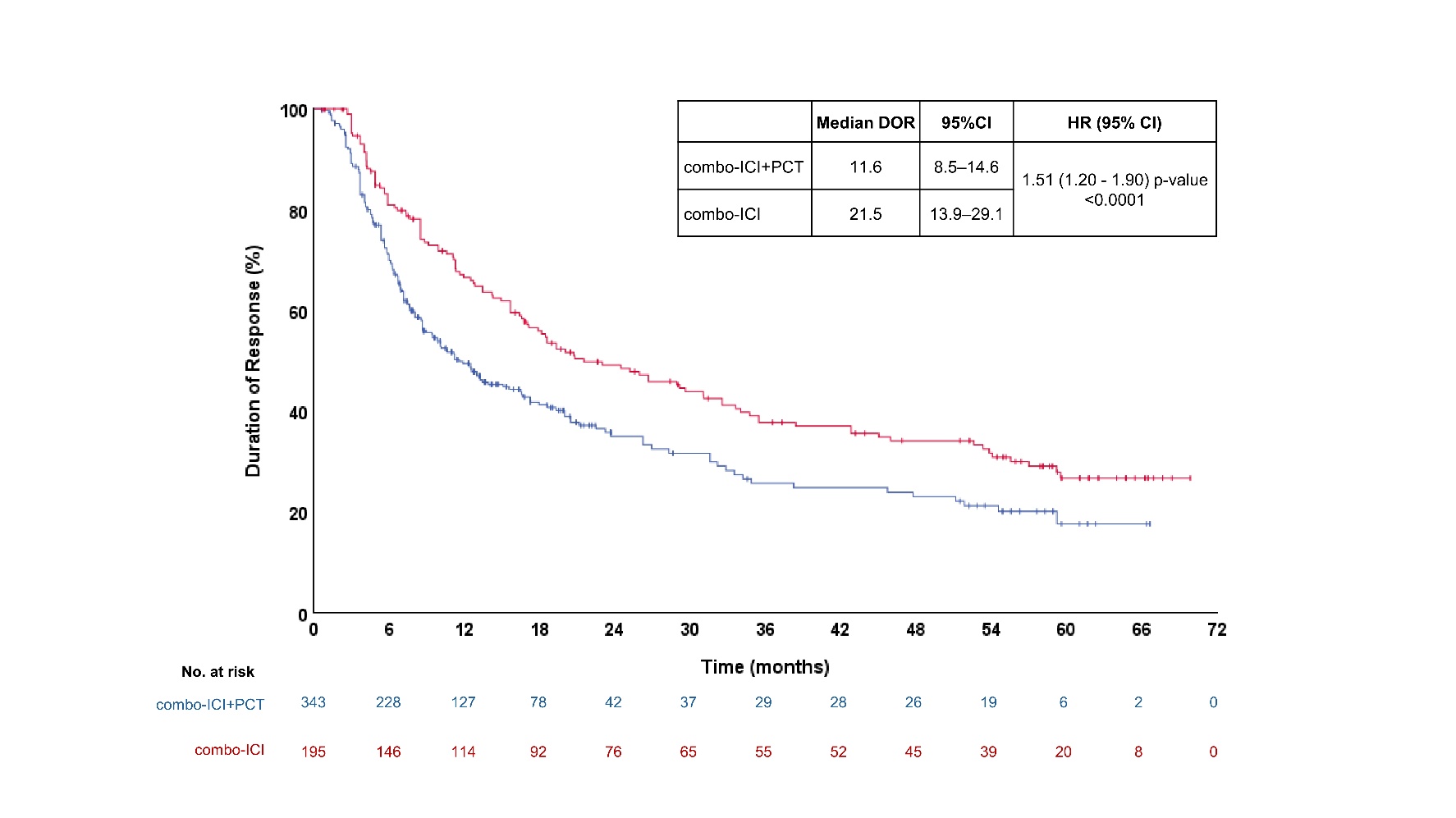

Supplement: Supplementary Figs. S1–S8 and Tables S1–S5 [file mmc1.docx]
